# Factors Influencing Carbon Stocks and Accumulation Rates in Eelgrass Meadows Across New England, USA

**Published:** 2020-12-01

**Authors:** A. B. Novak, M. C. Pelletier, P. Colarusso, J. Simpson, M. N. Gutierrez, A. Arias-Ortiz, M. Charpentier, P. Masque, P. Vella

**Affiliations:** 1 Earth and Environment, Boston University, Boston, MA, USA; 2 Atlantic Ecology Division, US EPA, ORD, NHEERL, Narragansett, RI, USA; 3 US EPA, Region 1, Boston, MA, USA; 4 MIT Sea Grant, Cambridge, MA, USA; 5 Institut de Ciència i Tecnologia Ambientals, Universitat Autònoma de Barcelona, Bellaterra, 08193 Barcelona, Spain; 6 Ecosystem Science Division, Department of Environmental Science, Policy and Management, University of California, Berkeley, CA, USA; 7 General Dynamics Corporation, Narragansett, RI, USA; 8 School of Science and Centre for Marine Ecosystems Research, Edith Cowan University, Joondalup, Western Australia 6027, Australia; 9 Departament de Física, Universitat Autònoma de Barcelona, Bellaterra, 08193 Barcelona, Spain; 10 International Atomic Energy, 4a Quai Antoine 1er, 98000 Principality of Monaco, Monaco; 11 Massachusetts Coastal Zone Management, Boston, MA, USA

**Keywords:** Seagrass, Blue carbon, Carbon sequestration, New England

## Abstract

Increasing the protection of coastal vegetated ecosystems has been suggested as one strategy to compensate for increasing carbon dioxide (CO_2_) in the atmosphere as the capacity of these habitats to sequester and store carbon exceeds that of terrestrial habitats. Seagrasses are a group of foundation species that grow in shallow coastal and estuarine systems and have an exceptional ability to sequester and store large quantities of carbon in biomass and, particularly, in sediments. However, carbon stocks (C_org_ stocks) and carbon accumulation rates (C_org_ accumulation) in seagrass meadows are highly variable both spatially and temporally, making it difficult to extrapolate this strategy to areas where information is lacking. In this study, C_org_ stocks and C_org_ accumulation were determined at 11 eelgrass meadows across New England, representing a range of eutrophication and exposure conditions. In addition, the environmental factors and structural characteristics of meadows related to variation in C_org_ stocks were identified. The objectives were accomplished by assessing stable isotopes of δ^13^C and δ^15^N as well as %C and %N in plant tissues and sediments, measuring grain size and ^210^Pb of sediment cores, and through assessing site exposure. Variability in C_org_ stocks in seagrass meadows is well predicted using commonly measured environmental variables such as grain size distribution. This study allows incorporation of data and insights for the northwest Atlantic, where few studies on carbon sequestration by seagrasses have been conducted.

## Introduction

The concentration of carbon dioxide (CO_2_) in the atmosphere has increased from 280 to 410 ppm since pre-industrial times and is expected to increase to 990 ppm by the end of this century. The accelerated increase of CO_2_ is primarily due to anthropogenic activities such as the burning of fossil fuels and the modification of land use for agriculture and deforestation (IPCC 2018). One strategy that has been proposed to mitigate for rising CO_2_ is to increase protection and restoration of coastal vegetated ecosystems (e.g., saltmarshes, mangroves, seagrasses; Mcleod et al. 2011; Howard et al. 2014). While coastal vegetated ecosystems comprise only 0.05% of the plant biomass on land, they store a comparable amount of carbon per year, making them one of the most important carbon sinks and mitigators of excess of CO_2_ on the planet (Smith 1981; Duarte et al. 2005; Nellemann et al. 2009; Mcleod et al. 2011).

Seagrasses are a group of foundation species that grow in shallow coastal and estuarine systems. They form extensive meadows, ranging from a few square meters to hundreds of square kilometers, and can be found along every continent except Antarctica (Green and Short 2003). Seagrass meadows provide many ecosystem services such as improved water quality and clarity, increased biodiversity and habitat, sediment stabilization, and nutrient accumulation (Orth et al. 2006). Like other vegetated coastal ecosystems, seagrasses also sequester and store large quantities of carbon in biomass and in sediments (Smith 1981; Duarte et al. 2005; Fourqurean et al. 2012; Röhr et al. 2018). Seagrass aboveground biomass is considered a short-term carbon sink and has a low contribution to the total carbon (C_org_) deposits found in meadows (Mateo et al. 2006; Fourqurean et al. 2012) due to exposure to aerobic conditions and herbivory (Enriquez et al. 1993; Mateo et al. 2006; Fourqurean et al. 2012). In contrast, sediments in seagrass meadows are considered a long-term carbon sink with large amounts of C_org_ deposits formed by refractory belowground biomass, seagrass detritus, as well as allochthonous C_org_ materials (e.g., marsh grass, macroalgae, benthic diatoms, phytoplankton, and seston; Gacia and Duarte 2001; Bouillon and Boschker 2006; Kennedy et al. 2010). Once the carbon accumulates in the sediment, it can remain there for decades to centuries due to anoxic conditions that inhibit microbial activity (Howard et al. 2014; Chmura et al. 2003; Trevathan-Tackett et al. 2017a). Recent assessments suggest that 300,000–600,000 km^2^ of the ocean is covered in seagrass habitats (0.1% of ocean surface), potentially storing between 4.2 and 8.4 Pg C, contributing to 10% of the annual carbon burial in the ocean (Duarte et al. 2005; Fourqurean et al. 2012).

Carbon storage (C_org_ stocks) and carbon accumulation rates (C_org_ accumulation) in seagrass meadows have been shown to vary spatially and temporally (Lavery et al. 2013; Samper-Villarreal et al. 2016; Gullström et al. 2018) and are influenced by a number of factors (see review by Mazarrasa et al. 2018). Biotic factors such as species composition, plant morphology (Lavery et al. 2013; Gillis et al. 2017), meadow structural complexity (Jankowska et al. 2016), and carbon origin (Mazarrasa et al. 2017) have been shown to influence C_org_ stocks in seagrass meadows. Abiotic factors such as water depth, wave height and exposure, and turbidity have also been shown to influence C_org_ stocks, with higher content at shallower depths, lower wave heights and exposures, and higher turbidities (Serrano et al. 2014; Samper-Villarreal et al. 2016; Mazarrasa et al. 2017). Elevated carbon in seagrass sediments has been associated with higher proportions of fine sediments, as well as with higher porosity, salinity, lower bulk density, and higher specific surface area (Röhr et al. 2016, 2018; Dahl et al. 2016; Gullström et al. 2018; Miyajima et al. 2017). While the effect of nutrient availability on C_org_ accumulation in sediments has been assessed through field and laboratory experiments, the relationship remains unclear (Armitage and Fourqurean 2016; Howard et al. 2016). In addition, the role of biotic and abiotic factors at different scales is poorly understood and critical to explaining interhabitat variability and estimating carbon budgets.

Eelgrass (*Zostera marina* L.) is the dominant seagrass species of north temperate oceans and a critical natural resource in coastal ecosystems. Along the east coast of the USA, it is estimated that up to 50% of all eelgrass habitat has been lost in the past century and the prospects for recovery in most of this area are low (Green and Short 2003; Neckles et al. 2009). The greatest anthropogenic threats have been eutrophication and sedimentation from urban and agricultural runoff (Short and Wyllie-Echiverra, 1996; Short et al. 2006; Waycott et al. 2009). Both eutrophication and sedimentation decrease the amount of light available to eelgrass for photosynthesis. Moreover, in systems with high nutrient loadings, epiphytes and fast-growing macroalgae outcompete eelgrass since they uptake nutrients more effectively and have relatively lower light requirements to sustain growth (Harlin and Thorne-Miller 1981; Short and Kaldy 1995; Twilley et al. 1985). Other anthropogenic activities having direct impacts on the distribution of eelgrass by reducing water clarity and/or uprooting plants include dredge and fill, land reclamation, and dock and jetty construction. The direct loss of eelgrass by organisms other than humans has also occurred through overgrazing (e.g., geese), bioturbation, and disease (Short and Wyllie-Echeverria 1996). Regardless of the cause, the loss of eelgrass results in the loss of ecosystem services they provide, including C_org_ sequestration, and potentially leads to CO_2_ emissions when sediment C_org_ deposits are eroded and exposed to aerobic conditions (Marba et al. 2015; Serrano et al. 2016a; Lovelock et al. 2017).

Despite the attention seagrasses have received as an important habitat for carbon storage, site-specific research is needed to understand how factors such as nutrient availability influence C_org_ stocks and C_org_ accumulation at various spatial scales. In this study, we contrasted C_org_ stocks, C_org_ accumulation rates, and sources of accumulated carbon in eelgrass meadows at 11 locations in New England representing a range of eutrophication and exposure conditions. Specifically, the objectives of our study were to quantify C_org_ stocks and C_org_ accumulation at local scales and for the region, as well as identify the environmental factors and structural characteristics of meadows related to variation in C_org_ stocks.

## Materials and Methods

### Study Area

**Fig. 1:**
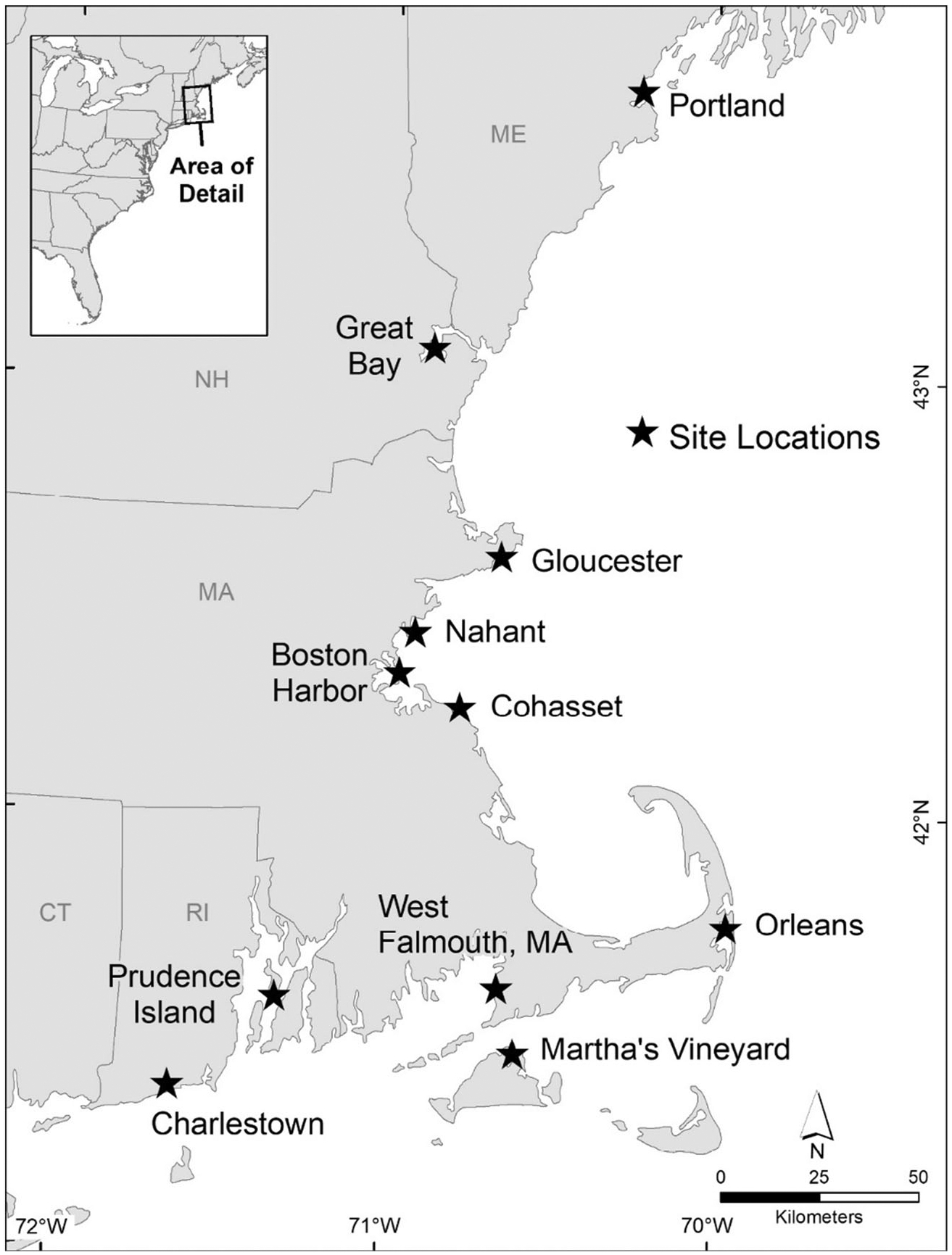
Map of study area

Eleven locations with persistent eelgrass meadows were selected from Maine to Rhode Island (Fig. 1, Table 1). A nutrient gradient was confirmed across sites using the methodology of Lee (2004; Fig. S1) with nitrogen in eelgrass leaves ranging from 0.93 to 2.44%. Sites also represented a range of physical exposure: Great Bay, Orleans, West Falmouth, and Charlestown are fully enclosed embayments: Nahant and Cohasset are exposed coastal sites, and the remaining sites have some partial enclosure. With the exception of Great Bay, all sites have generally high salinity (29–32), although there may be intermittent freshwater inputs. Sediment texture ranges from silt to medium sand. Tides range from 0.55 m at sites from Cohasset and north to 1.13 m from Orleans and south (Table 1). All sites except Portland Harbor, Boston Harbor, and Prudence Island were sampled during the summer of 2016. The remaining three sites were sampled during the summer of 2017. Samples were collected from the middle of each meadow, at least 6 m from the meadow’s edge in all directions. Water column depths ranged from 1 to 2 m, with the exception of Gloucester, which was approximately 5 m. Samples were also collected outside of each meadow in an unvegetated location.

### Field Collection

#### Sediment Traps

Sediment traps were deployed at each site to provide insights into the amount and type of material being delivered to the meadow and unvegetated site. Six sediment trap arrays, with three traps per array, were deployed for 14 days at the sediment surface at each site to quantify sediment deposition. At each of the 11 sites, three arrays (Fig. S2) were placed inside the meadow and three arrays were placed outside the meadow on bare substrate. Each trap consisted of a PVC tube (length 15 cm, inner diameter 5 cm). A honeycomb baffle (Plascore, Zeeland, MI) was placed inside each trap to prevent resuspension of particles.

#### Sediment Cores

Nine sediment cores (five within the meadow and four from adjacent unvegetated sites outside of the meadow) were taken at each site. A 50-cm core barrel (diameter 7 cm) was manually driven to the point of refusal using a core head with a T handle and check valve. Cores were extracted, capped at both ends under water, and kept in a vertical position during transport to shore. Compaction during sampling was observed in 7 of the sites and was assessed by measuring the depth to the sediment surface inside and outside of the core. The depth of refusal averaged 30 cm (decompressed) except for Charlestown and Great Bay where it was not reached. Carbon stock estimates were then normalized to 30 cm which is close to the depth of refusal in most of the sites, thus include the full range of seagrass organic carbon deposits. Eight of the cores (four meadow and four unvegetated) were divided into sections (0–2 cm, 2–5 cm, 5–10 cm, 10–20 cm, 20–30 cm). Six of the cores were used to measure dry bulk density, sediment organic matter (OM) content (as loss on ignition), %C, %N, and stable isotopes of δ^13^C and δ^15^N while two of the cores (one within and one outside of the meadow) were used to measure grain size. The final core from within the meadow was extruded into 1-cm sections from which a 10–15-g subsample was taken from each section for ^210^Pb analysis and age determination. In these cores, dry bulk density and %C and %N were determined at 2-cm intervals.

#### Eelgrass

Shoot density of each meadow was determined by randomly tossing a 0.0625-m^2^ quadrat (*n* = 5) within a 4-m radius of the sediment traps at each site. Shoot density was estimated in situ by counting the number of shoots within each quadrat.

For morphological measurements, 21 to 30 representative shoots, consisting of both aboveground and belowground material, were collected within a 3-m radius of the sediment traps at each site. An additional 10 eelgrass shoots were haphazardly collected from the middle of the meadow at each site for analyses of δ^13^C, δ^15^N, %C, and %N content.

Growth of individual leaves was determined using the leaf marking techniques described by Short (1987). Thirty shoots located within the vicinity of sediment traps were haphazardly selected at each site except Boston Harbor and marked by making a pinhole with a safety pin through the leaf sheath. Fourteen days after initial marking, the shoots were harvested and the distance between the pinhole on each leaf and the residual scar on the sheath was measured along with leaf width, leaf length, and the dried leaf weight of the youngest fully mature leaf. If a young leaf did not have a pinhole, it was considered new growth. The total area of new tissue added per shoot was divided by the number of days, and a linear relationship was developed between leaf weight and length (Short and Duarte 2001). Shoot growth rate is expressed as mg dry weight/shoot/day and cm/shoots/day. Marked shoots from Martha’s Vineyard could not be found and were, therefore, not assessed.

### Laboratory Analysis

#### Sediment Traps

After field deployment, the sediment traps were capped, returned to the laboratory, and allowed to settle for 4 h at room temperature. The baffles were then removed, the overlaying water was drained, and the three traps in each array were combined and decanted into one beaker. Fresh vegetation, shells, or small living organisms (fish, shrimp, etc.) were removed from the sample. The sediment was then transferred to a pre-weighed pan and dried to a constant weight for 48 h at 60 °C. After the sediment was dried to a constant weight, the weight was determined. The material was then crushed into a fine powder and analyzed for δ^13^C and ^δ15^N as well as carbon (%C) and nitrogen (%N) content (see “Stable Isotope Analysis” section below). Sediment traps for Boston Harbor were lost and were, therefore, not analyzed.

#### Sediment Cores

Dry bulk density of each sediment core section was determined using the mass of sediments dried at 60 °C for 7 days until they reach a constant weight and then divided by the volume of the sediment section. Following bulk density measurements, the sample was subdivided using a sediment splitter to obtain a smaller portion for stable isotope and organic matter analyses. Shells as well as large rhizomes and roots were removed from the subsample as the goal of this study was to quantify only carbon accumulation from deposition and fragmentation of autochthonous and allochthonous materials (Greiner et al. 2013). The remaining material was homogenized by grinding to a fine powder.

For samples collected in 2016, each processed sample was divided into two subsamples. The first was analyzed for δ^13^C, δ^15^N, %C, and %N, while the second was placed in a muffle furnace at 450 °C for 16 h to determine %OM using the loss on ignition method (Howard et al. 2014). Studies of C_org_ stocks have used both %C and organic matter determined using loss on ignition, so to facilitate comparison across studies, both techniques were applied in 2016. In 2017, only δ^13^C, δ^15^N, %C, and %N were measured.

Grain size was determined for each sediment core section using the Malvern Mastersizer 2000 with the Hydro 2000S wet dispersion unit (Malvern Instruments, Malvern, UK) system. Sediment samples were homogenized and extruded through a 2-mm sieve into a beaker; then, deionized water was added to the sample to create a suspension that was then analyzed.

#### Eelgrass

The morphological samples were rinsed with deionized water and thoroughly cleaned of epiphytes prior to counting the number of leaves per shoot and measuring leaf length (cm), leaf width (cm), and the distance between nodes on the rhizome (internode length; cm). Aboveground and belowground material on each shoot was then separated, dried for 48 h at 60 °C until it reached a constant weight, and weighed to determine the aboveground and belowground weight (g) of each shoot. The first fully mature leaf from each shoot collected for chemical analysis was cleaned, dried, and ground to a fine powder before analysis.

#### Stable Isotope Analysis

Stable isotope analyses of δ^13^C and δ^15^N, as well as measurements of %C and %N, were measured in plant and sediment samples using the Isoprime 100 isotope ratio mass spectrometer (IRMS) interfaced with a Micro Vario Elemental Analyzer (Elementar Americas, Mt. Laurel, NJ). Sediment trap, core, and eelgrass samples were weighed into 6 × 4 mm tin capsules (10–15 mg, 25–30 mg, and 4–6 mg, respectively). Due to low amounts of nitrogen in the core sediments, samples were double-tinned when necessary (tin acting as a catalyst for a greater combustion in the IRMS). Internal laboratory standards were used throughout runs, every 15–20 samples, to account for instrument offset (BCSS-1 for sediments and blue mussel homogenate for eelgrass). The %C in seagrass leaves was based on 3 composite samples.

#### Core Dating Analysis

^210^Pb was determined through the analysis of ^210^Po by alpha spectrometry after addition of ^209^Po as an internal tracer and digestion in acid media using an analytical microwave (Sanchez-Cabeza et al. 1998). The concentrations of excess ^210^Pb used to obtain the age models were determined as the difference between total ^210^Pb and ^226^Ra (supported ^210^Pb). Concentrations of ^226^Ra were determined for selected samples along each core using two methods: the ultra-low-level liquid scintillation spectrometer Wallac 1220 Quantulus (PerkinElmer, Waltham, MA) using a technique adapted from Masqué et al. (2002) and gamma spectrometry through the emission lines at 295 and 352 keV of its decay product ^214^Pb using calibrated geometries in a HPGe detector (CANBERRA, Mod. SAGe Well). The CF/CS model was used to calculate mean sedimentation rates over the last 100 years at all sites (Krishnaswami et al. 1971; Arias-Ortiz et al. 2018a). The CRS model (Appleby and Oldfield 1978) was also used to calculate sedimentation rates at Niles Beach. This model could not be applied to the other cores because excess ^210^Pb was not analyzed for all sections and/or the horizon of excess ^210^Pb was not reached (i.e., the sediment cores were too short). C_org_ accumulation was calculated by multiplying the average mass accumulation rate by the weighted average of C (% dry weight) content of the dated period.

### Calculations

#### Carbon Stock

C_org_ stocks in the sediment traps were calculated by multiplying the %C by the amount of material in the sediment traps and dividing by the sediment trap surface area. The depth-integrated C_org_ stocks were calculated according to Lavery et al. (2013) by multiplying the %C measured along the sediment core by the corresponding dry sediment bulk density (g/cm^3^). These numbers were then depth-integrated over the core length to estimate C_org_ stocks. C_org_ stocks were normalized to a depth of 30 cm similar to the average depth of refusal. The eelgrass carbon stock was determined by multiplying %C by the aboveground eelgrass weight (g/shoot) by shoot density.

#### Physical Exposure

Physical exposure was determined by calculating relative wave energy (RWE) using WEMo (wave exposure model; https://coastalscience.noaa.gov/research/coastal-change/wemo/), and by calculating degree of sorting (Folk and Ward 1957), a proxy for exposure (Röhr et al. 2018) based on the grain size distribution. WEMo uses linear wave theory to calculate actual wave height and derived wave energy while taking into consideration wind generation and local water depth characteristics such as shoaling and dissipation from breaking waves. WEMo modeling was performed using present default value conditions as specified in Fonseca and Malhotra (2010).

### Statistical Analysis

#### Determination of Differences Among Sites

Morphological and structural variation across eelgrass populations was analyzed using a multivariate analysis of variance (MANOVA). The MANOVA indicated significant differences among populations as well as multicollinearity. Three dependent variables (leaf length, leaf width, and aboveground weight) had a partial correlation above 0.7 with leaf area, and one dependent variable (belowground weight) had a partial correlation with internode length. The four dependent variables were removed before conducting individual analyses of variance (ANOVAs) on the remaining dependent variables (number of leaves, internode length, leaf area, aboveground to belowground weight) followed by Tukey’s post hoc tests using JMP. One-way ANOVA was also conducted on shoot density and growth measurements.

Two-way ANOVA was used to analyze differences between sites in carbon stocks (g C/m^2^) in sediment traps and sediment cores using SAS (version 9.4, SAS Institute Inc.). Carbon stocks in the aboveground eelgrass could not be compared statistically because shoot density, morphology, and carbon content were not collected from the same sample. Among site comparisons for plant morphology, meadow structure, and growth were made using one way-ANOVA and Tukey’s post hoc tests using JMP (version 12.1, SAS Institute Inc.). All datasets met the assumptions of equal variance according to the Browne-Forsythe test. Values are reported as means and standard errors.

#### Relating Environmental Variables to Carbon Stocks

Multimodel inference (Burnham and Anderson 2002) was used to investigate the relationship between carbon stocks and various environmental variables. In this approach, multiple models are created using all possible combinations of the independent variables thought to be important to the variable being modeled. Linear regression models were generated using SAS 9.4. The carbon stocks in the top 5 cm of the core (the root zone) were predicted, with separate models for the meadow cores and unvegetated cores.

Because carbon stocks appeared to be related to latitude, variables related to latitude that were also thought to impact seagrass meadows were assembled. Tidal range was obtained from NOAA datums (https://tidesandcurrents.noaa.gov/stations.html?type=Datums). Water temperature was obtained from EPA’s National Coastal Assessment Program (http://www.epa.gov/emap). Insolation was obtained from NASA (https://power.larc.nasa.gov/data-access-viewer/). All three variables were significantly correlated with latitude but because water temperature was significantly related to tidal range (*r* = − 0.596, *p* = 0.003, Table S1), only insolation and tidal range were retained for later modeling. The seven potential environmental variables were assembled to relate to bulk carbon in the sediments including tidal range, insolation, degree of sorting, relative wave energy, %silt-clay, mass in the sediment trap, and %N in the sediment trap. For the meadow sites, %N in eelgrass leave was also considered. Salinity was not included because the mean salinity range at our sites was low, most within 3 PSU. To reduce the possibility of multicollinearity, the correlation among variables was considered separately for the meadow (Table S2) and unvegetated (Table S3) sites. For the meadow sites, the six independent variables retained for inclusion in models were tidal range, insolation, degree of sorting %silt-clay, mass in the sediment trap, and %N in the sediment trap (Table S4). For the unvegetated sites, the four independent variables retained for inclusion in models were tidal range, relative wave energy, %silt-clay, and mass in the sediment trap (Table S4). For the meadow sites, an additional set of models were constructed that included eelgrass morphology and growth. Because leaf area and growth were significantly correlated (*r* = 0.667, *p* = 0.0499), only growth was retained. Growth was also significantly correlated with %N in the sediment trap (*r* = − 0.669, *p* = 0.049), while shoot density was significantly correlated with both insolation (*r* = 0.663, *p* = 0. 037) and mass in the sediment trap (*r* = − 0.808, *p* = 0.005). The final meadow models with both environmental and eelgrass variables included five variables: tidal range, degree of sorting, %silt-clay, shoot density, and growth (Table S4). Because Boston Harbor was missing both growth and sediment trap data, it was not included in any of these models.

Relative importance of individual variables was determined examining the sum of the weighted Akaike’s information criterion (AICω) for every model containing that variable (Burnham and Anderson 2002). ICω are calculated as AICω = exp. (− 0.5 × ΔAIC individual model) / ∑ exp. (− 0.5 × ΔAIC all models) where ΔAIC = AIC individual model − AIC min. A final model average was obtained by multiplying the coefficients for a given model by the AICω for that model, and then averaging the resulting coefficients for a given variable across all models. To see if a more parsimonious model might be possible, a model using only the most important variables based on ∑AICω was compared with the full model with all variables using the likelihood ratio statistic, *G*^2^ (Agresti 1996). The best, most parsimonious submodel was selected that had a low AIC value and a non-significant *G*^2^ test.

## Results

### Eelgrass Morphology, Structure, and Growth

**Fig. 2:**
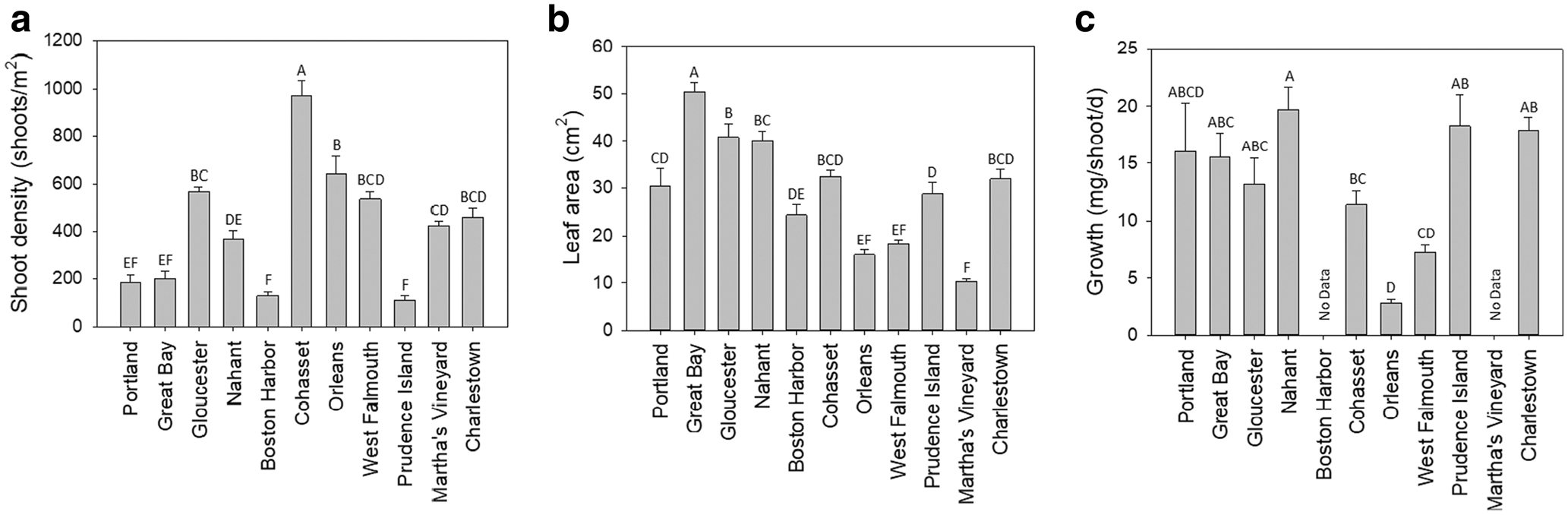
Structural, morphological, and growth characteristics of eelgrass meadows. Letters indicate statistical differences among sites. **a** Shoot density (shoots/m^2^). **b** Leaf area (cm^2^). **c** Growth (mg/shoot/day)

Univariate comparisons using one-factor ANOVA showed significant differences among sites for structural, morphological, and growth characteristics (Fig. 2a, shoot density *F*_10,44_ = 44.4924, *p* < 0.0001; Fig. 2b, leaf area *F*_10, 246_ = 42.773, *p* < 0.0001; Fig. 2c, growth mg/shoot/day *F*_8125_ = 11.0637, *p* < 0.0001; Fig. S3a, leaves/shoot *F*_10, 246_ = 11.1510, *p* < 0.0001; Fig. S3b, internode length *F*_10, 241_ = 19.8383, *p* < 0.0001; Fig. S3c, aboveground-belowground weight *F*_10, 245_ = 7.5289, *p* < 0.0001; Fig. S3d, growth cm/shoot/day *F*_8125_ = 33.3469, *p* < 0.0001). Cohasset Harbor had the highest shoot density while Boston Harbor and Prudence Island had the lowest (equivalent to Portland and Great Bay). Great Bay shoots had the greatest leaf areas (Fig. 2b), internode lengths (equivalent to Prudence Island; Fig. S3), and growth rates cm/shoot/day (equivalent to Portland; Fig. S3). Charlestown shoots had more leaves than all sites except Nahant (Fig. S3).

### Carbon Stocks

**Fig. 3:**
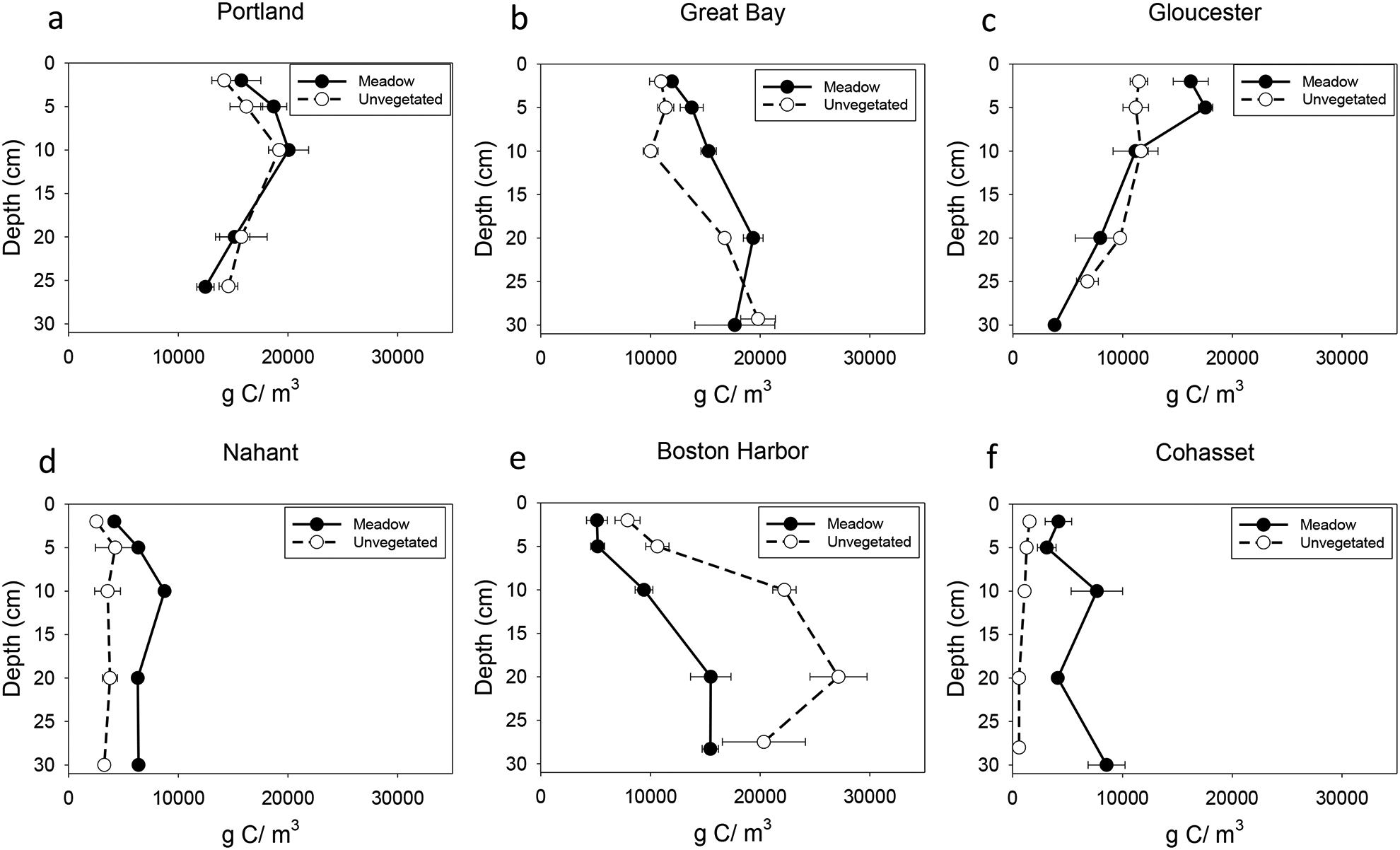

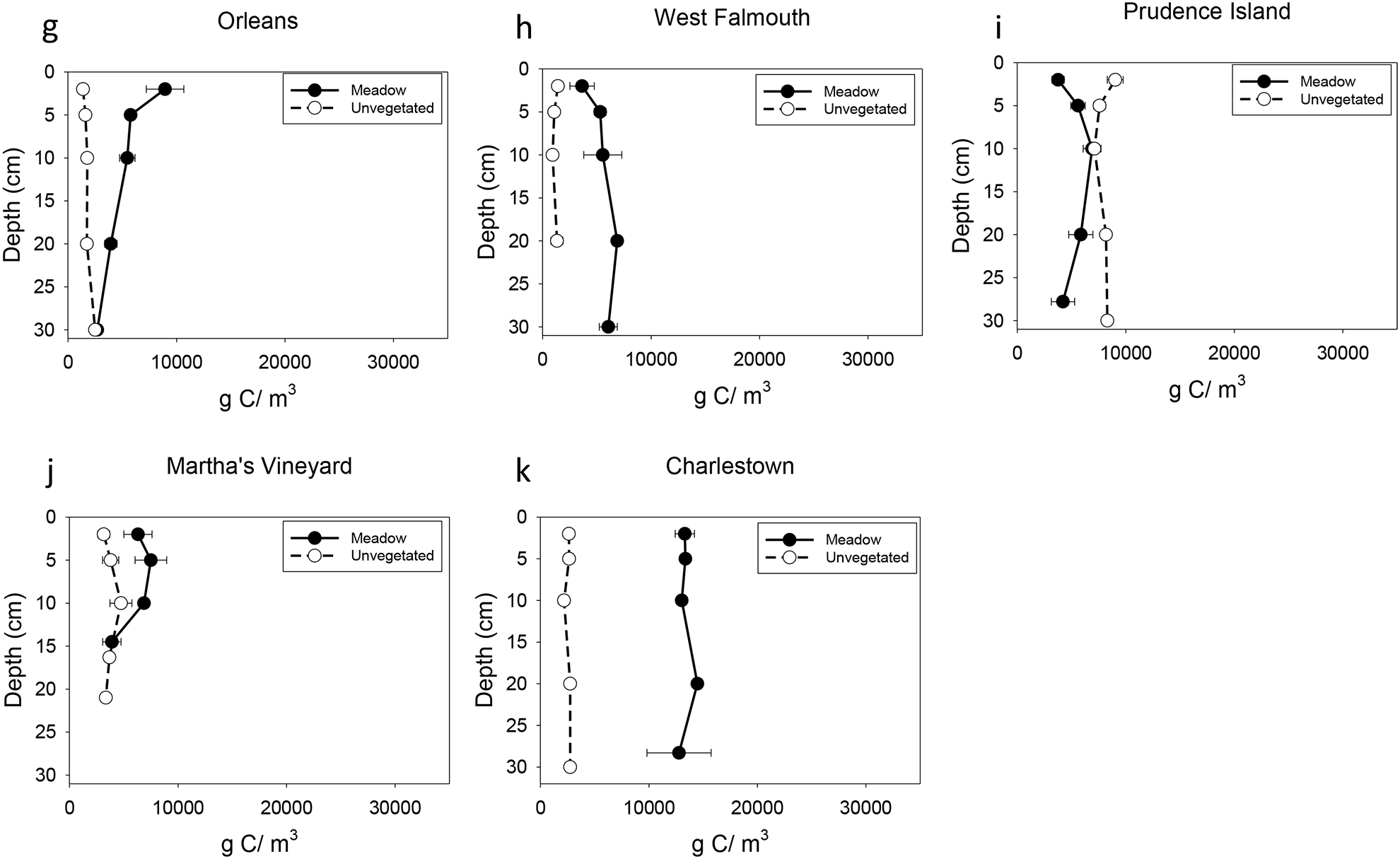
Depth profiles of carbon density. Meadow sites are indicated by a closed black symbol and solid line, while the unvegetated sites are indicated by an open symbol and dotted line. **a** Portland, ME. **b** Great Bay, NH. **c** Gloucester, MA. **d** Nahant, MA. **e** Boston Harbor, MA. **f** Cohasset, MA. **g** Orleans, MA. **h** West Falmouth, MA. **i** Prudence Island, RI. **j** Martha’s Vineyard, MA. **k** Charlestown, RI

Carbon depth profiles varied by site (Fig. 3). Distinct subsurface peaks were seen in Portland and Boston Harbor. Some declines in carbon density with depth were observed especially in meadow sites in Gloucester, Orleans, and Martha’s Vineyard. Assessment of C_org_ stocks in this study were based on %C from the IRMS output; however, we found a tight correlation between %C from the IRMS and organic matter assessed using loss on ignition (adj *R*^2^ = 0.98), allowing translation between methods (Fig. S4).

Sediment trap C_org_ stocks ranged from 30 to 550 g C/m^2^ in meadows and from 70 to 500 g C/m^2^ in unvegetated areas. Significant differences were observed among sites (*F*_9,59_ = 179.78, *p* < 0.0001), among treatments (meadow vs unvegetated sites; *F*_9,59_ = 69.43, *p* < 0.0001), and a significant site per treatment effect (*F*_9,59_ = 23.93, *p* < 0.0001). Most unvegetated sites had higher carbon (g C/m^2^) in sediment traps in unvegetated areas than those in the meadow sites; only three meadow sites had higher carbon (g C/m^2^) in sediment traps relative to traps located in unvegetated areas (Fig. S5).

**Fig. 4:**
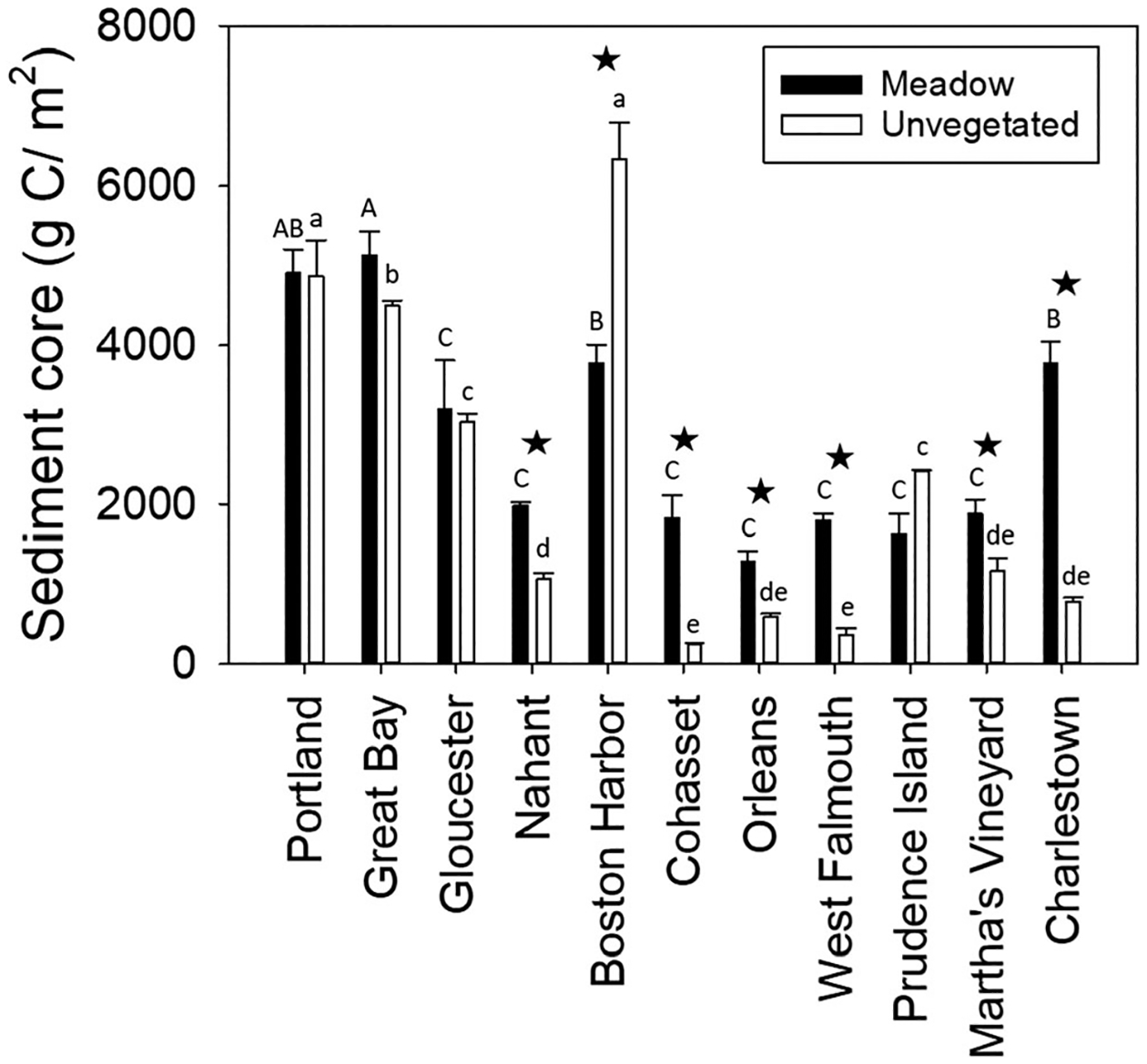
Sediment carbon stock (g C/m^2^) from cores collected in meadow and unvegetated areas. Capital letters indicate statistical differences among sites in meadows, while lowercase letters indicate statistical differences among sites in unvegetated areas. Stars indicate significant differences between meadow and unvegetated areas

**Fig. 5:**
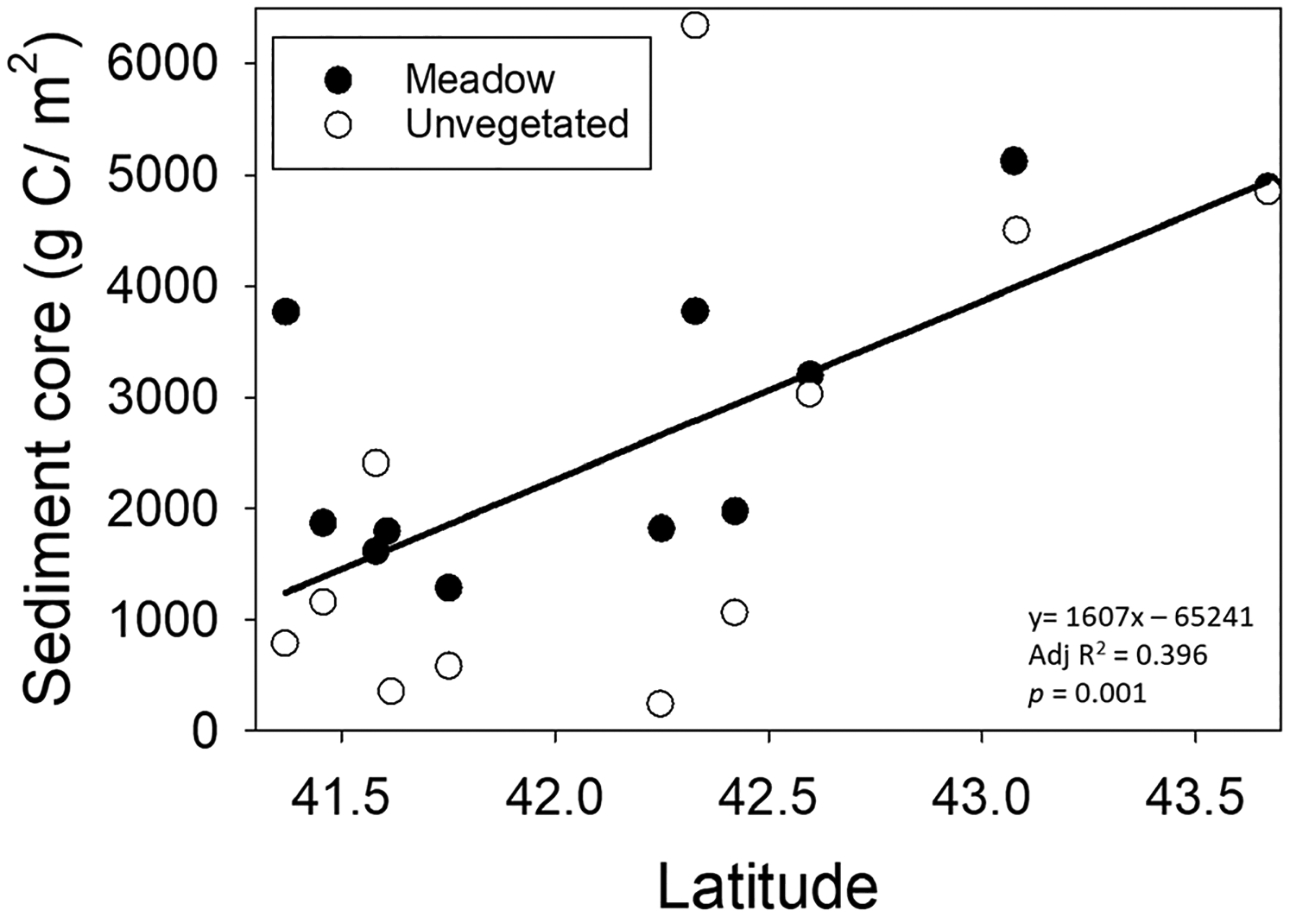
Scatter plot showing significant increases in sediment carbon stocks with increasing latitude. The symbols represent the site mean for all 11 sites

C_org_ stocks in the upper 30 cm of the sediments ranged from 1500 to 4500 g C/m^2^ in meadows and from 100 to 5500 g C/m^2^ in unvegetated sites and were significantly different across sites (*F*_10,65_ = 84.76, *p* < 0.0001) and treatments (*F*_1,65_ = 24.33, *p* < 0.0001). There was also a significant site per treatment interaction (*F*_10,65_ = 15.78, *p* < 0.0001); nine of the 11 sites had higher C_org_ stocks in sediment cores from meadows relative to those from unvegetated sites (Fig. 4). Portland, Great Bay, Boston Harbor, and Charlestown had the highest C_org_ stocks. The apparent north–south gradient in C_org_ stocks was statistically significant (Fig. 5).

**Fig. 6:**
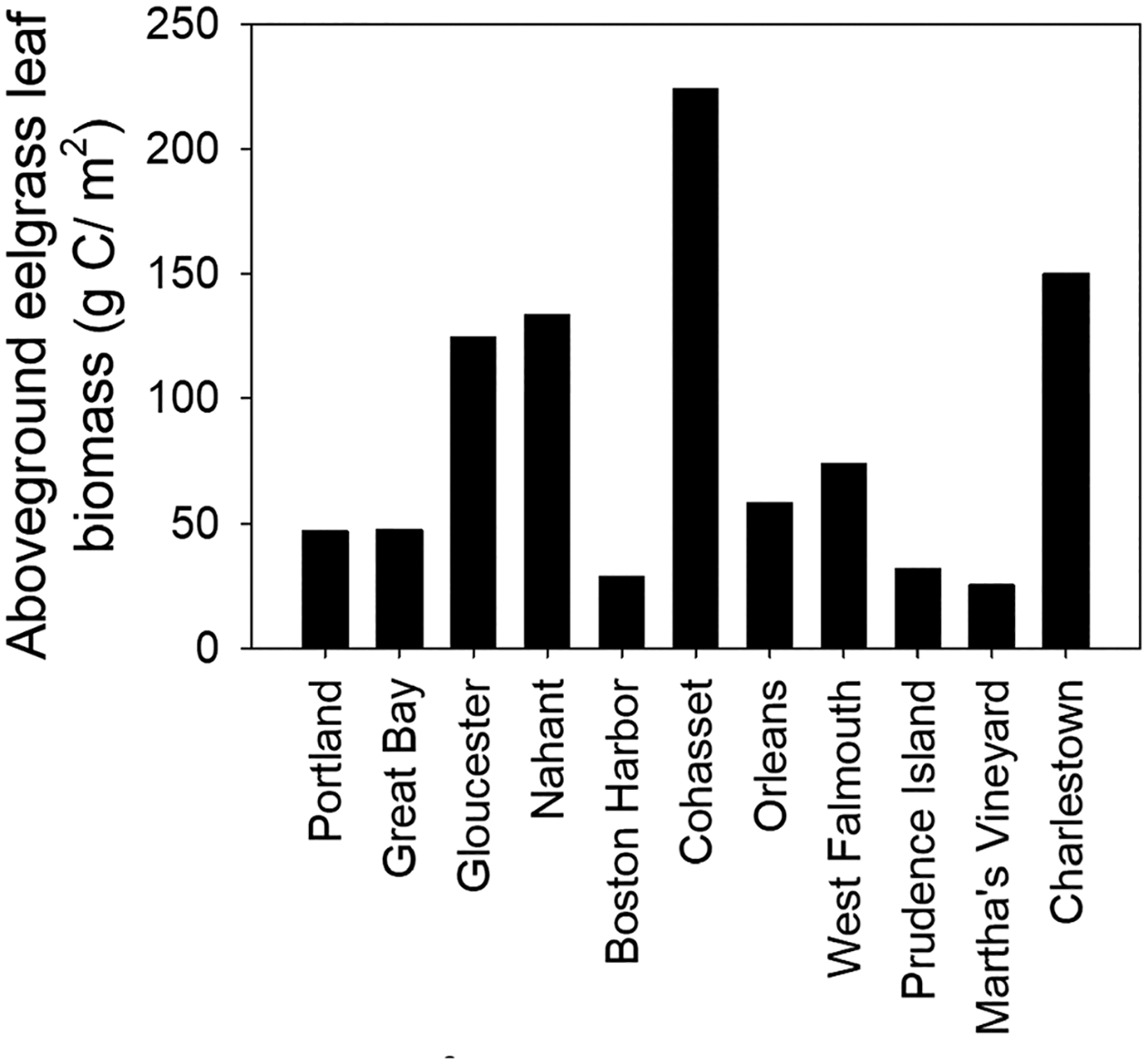
Carbon stock (g C/m^2^) of aboveground eelgrass across study sites

Because of the lack of synoptically collected samples, we were not able to perform statistical analyses of the carbon stock in aboveground vegetation. However, based on visualizing the data, there were apparent differences among sites (Fig. 6), and there was no significant relationship between biomass carbon stocks and latitude (*p* = 0.897).

For the meadow sites, 126 possible models were generated between carbon stocks in the top 5 cm of the core and tidal range, insolation, degree of sorting, %silt-clay contents, mass in the sediment trap, and %N in the sediment trap. Sixty-two models were generated between carbon stocks in the top 5 cm of the core and tidal range, degree of sorting, %silt-clay, shoot density, and growth. In the unvegetated areas, 30 models were generated between carbon stocks in the top 5 cm of the core and were tidal range, relative wave energy, %silt-clay, and mass in the sediment trap.

The AICω for each model was used to calculate the relative importance of the individual variables. For meadow cores, only considering environmental variables, %silt-clay had the highest importance (∑AICω = 0.77) in predicting the C_org_ stocks in the top 5 cm of the core, while %N in the sediment trap had the next highest importance (∑AICω = 0.39) and the remaining variables had lower importance (Table 2). When eelgrass variables were included, the pattern changed. Degree of sorting had the highest importance (∑AICω = 0.82), shoot density also had high importance (∑AICω = 0.74), %silt-clay having moderate importance (∑AICω = 0.44), and tidal range (∑AICω = 0.16) and growth (∑AICω = 0.12) had lower importance (Table 2). In the unvegetated areas, %silt-clay had the highest importance (∑AICω = 1.00), while the remaining variables had low importance in predicting the carbon stock in the top 5 cm of the core (Table 2).

**Fig. 7:**
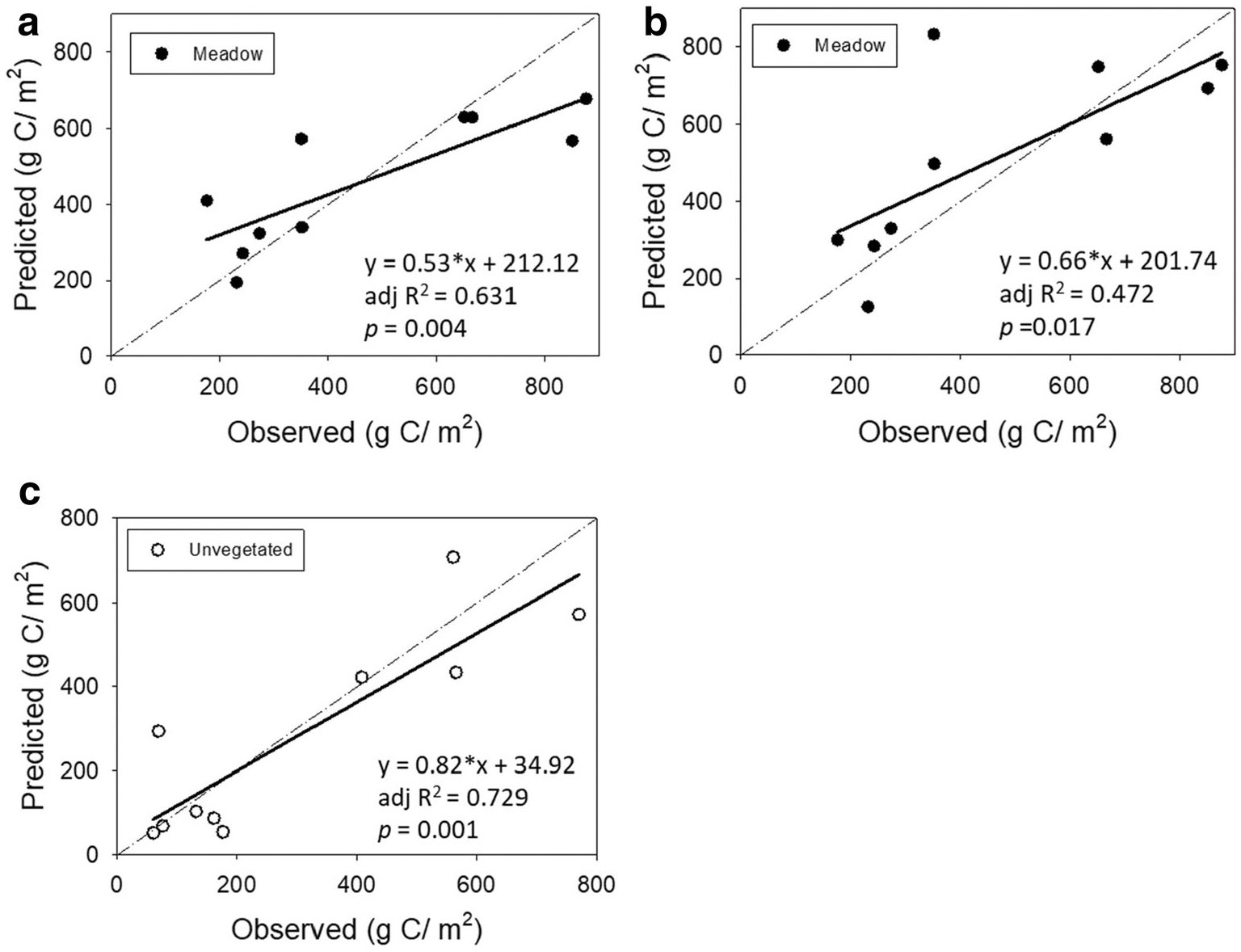
Predicted *C*_org_ stocks in sediments using environmental and eelgrass variables. The dashed line indicates a 1:1 relationship between the observed data and the modeled output. The solid line shows the regression line that best fits the predicted output. For the regression equation, *y* = predicted bulk carbon (g C/m^2^), *x* = observed bulk carbon (g C/m^2^), and adj *R*^2^ is the amount of variance explained after correcting for the number of parameters in the model. **a** Model output for top 5 cm of core in meadow, predicted using environmental variables only. The figure shows model average results based on 126 models and 10 sites. **b** Model output for top 5 cm of core in meadow, predicted using a combination of environmental and eelgrass variables. The figure shows model average results based on 62 models and 10 sites. **c** Model output for top 5 cm of core in unvegetated areas predicted using environmental variables only. The figure shows model average results based on 30 models and 10 sites

The final averaged model based on environmental variables alone at meadow sites was able to significantly predict (adj *R*^2^ = 0.631, *p* = 0.004) carbon stock in the top 5 cm of the core (Fig. 7a). When including eelgrass variables, predictive ability decreased (adj *R*^2^ = 0.472) but was still significant (*p* = 0.017; Fig. 7b). The final averaged model at unvegetated sites was also able to significantly predict (adj *R*^2^ = 0.729, *p* = 0.001) the carbon stock in the top 5 cm of the core (Fig. 7c). In the meadow sites, using only the variables with the highest importance (degree of sorting, shoot density, and %silt-clay) resulted in a model that performed as well as the saturated model (tidal range, degree of sorting, %silt-clay, shoot density, and growth) as indicated by a lower AIC value and non-significant *G*^2^ test. However, the amount of variance explained was slightly lower (adj *R*^2^ = 0.418), although still significant (*p* = 0.026; Fig. S6a). In the unvegetated areas, sediment grain size distribution alone could predict C_org_ stocks in the top 5 cm (adj *R*^2^ = 0.728, *p* = 0.001) as well as the full model (Fig. S6b), as indicated by a lower AIC value and non-significant *G*^2^ test.

### Carbon Source, Carbon Accumulation, and Age of Cores

**Fig. 8:**
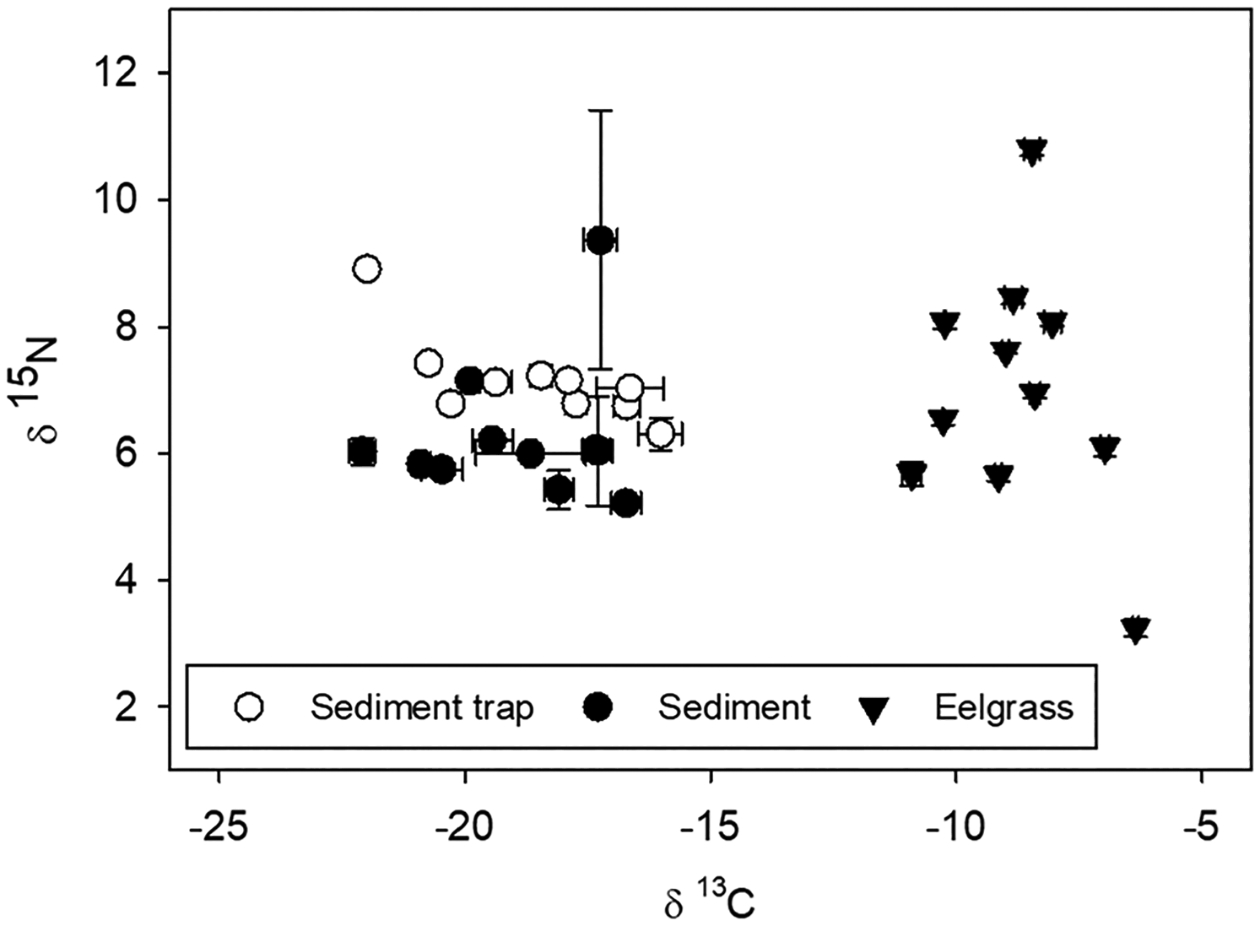
Biplot showing isotopic signal of material from sediment traps, sediment from cores and aboveground eelgrass. The symbol is the mean value while the error bars are standard errors

Isotopic biplots were used to investigate the source of material to the sediments in the meadow (Fig. 8). Sediment trap material δ^13^C ranged from − 22.13 to − 15.10 ‰, and δ^13^C values in sediment were similar to the sediment trap material, ranging from − 22.93 to − 14.86 ‰. δ^15^N values of sediment trap material ranged from 5.78 to 9.13 ‰, while δ^15^N in the sediment had a much broader range (3.90 to 17.81 ‰). Aboveground eelgrass had a much different isotopic signature than the sediment trap material or the sediment (Fig. 8). δ^15^N of eelgrass ranged from 3.04 to 9.13 ‰; δ^13^C ranged from − 12.24 to − 6.26 ‰.

Cores from Boston Harbor, West Falmouth, and Martha’s Vineyard could not be dated with the ^210^Pb method because of either significant mixing or because sediment accumulation was too low or negligible. Great Bay showed the highest sedimentation and C_org_ accumulation rates (1.19 ± 0.15 g/cm^2^/year and 230 ± 30 g C/m^2^/year, respectively), over the dated period which encompassed the last two decades (18 years in the upper 30 cm, Table 3). This contrasts with the Gloucester, MA site that has the lowest sedimentation rate (0.12 ± 0.01 g/cm^2^/year) and Orleans or Prudence, which showed the lowest C_org_ accumulation rates (8–9 g C/m^2^/year) in the upper 30 cm of the core over the last century (Table 3).

## Discussion

Eelgrass is the most wide-ranging seagrass species in the Northern Hemisphere and provides important ecosystem services to coastal environments. Recent studies have shown significant variation in estimates of carbon stocks (C_org_ stocks) and carbon accumulation rates (C_org_ accumulation) in seagrass meadows, suggesting that more information is needed to understand the factors influencing variability (Fourqurean et al. 2012; Lavery et al. 2013; Röhr et al. 2018). In our study, we estimated C_org_ stocks and C_org_ accumulation at 11 eelgrass meadows across New England, representing a range of eutrophication and exposure conditions. In addition, we identified the environmental factors and structural characteristics of meadows related to variation in C_org_ stocks. Our results show large variation in C_org_ stocks and C_org_ accumulation across New England eelgrass meadows (Table 4).

### Carbon Stocks in Eelgrass Meadows

Overall most of the New England eelgrass meadows in this study had higher sediment C_org_ stocks than nearby unvegetated areas (Fig. 4), supporting previous studies that indicate seagrass ecosystems are a significant carbon sink (Fourqurean et al. 2012; Röhr et al. 2018). The exceptions were primarily at sites with higher nutrients, where allochthonous carbon accumulated in both meadow and unvegetated sediments equally. The average sediment C_org_ stock in the upper 30 cm for eelgrass in New England (2832 ± 416 g C/m^2^) was comparable to worldwide estimates for eelgrass (2721 ± 989 g C/m^2^; Röhr et al. 2018), but lower than global estimates that include all seagrass species (19,420 ± 202 g C/m^2^; Fourqurean et al. 2012; Table 4). The highest C_org_ stocks occurred in Portland Harbor and Great Bay eelgrass meadows. At both sites, sediment C_org_ stocks were twice as high as worldwide estimates for eelgrass and three times as high as the average C_org_ stocks for eelgrass meadows in the Atlantic Ocean, Pacific Ocean, Baltic Sea, and Black Sea (Röhr et al. 2018). However, this was also the case for their concurrent bare analogues, suggesting that C_org_ storage at these sites is driven by local nutrient loading and depositional environment. In addition to having high C_org_ stocks, meadows in Portland Harbor, Great Bay, as well as Charlestown had the highest C_org_ accumulation rates. Excluding sites with higher nutrients, eelgrass C_org_ stocks were highest in Charlestown, being two times higher than average C_org_ stocks found in temperate eelgrass meadows globally (Röhr et al. 2018). Carbon content in aboveground biomass was also estimated at each site and the average (86 ± 19 g C/m^2^) for New England was comparable to estimates for all species in the Temperate North Atlantic Bioregion (Table 4; i.e., *Ruppia maritima*, *Z. marina*, *Z. noltii*, *Cymodocea nodosa*, and *Halodule wrightii*; Short et al. 2007; Fourqurean et al. 2012).

### Factors Influencing C Stocks

Our study was initiated under the assumption that differences in eutrophication and exposure would be reflected in the amount of carbon stored within eelgrass meadows. The results provided some support for the role of these parameters in carbon storage. Our multimodel inference model using environmental variables showed that % nitrogen in sediment traps was of secondary importance in eelgrass meadows (Table 2). In addition, sites with the highest nutrient levels (Portland, Great Bay) had the highest C_org_ stocks in both meadow and unvegetated areas (Table 1; Fig. 4). Other studies have also shown that nutrient inputs can influence carbon storage and accumulation in seagrass meadows as well as in unvegetated sediments due to an increase in the rate of organic matter supplied to the system (Gacia et al. 2002; Mazarrasa et al. 2017; Samper-Villarreal et al. 2017; Kindeberg et al. 2018). Moreover, increasing organic matter can influence morphology and structure of eelgrass meadows so that carbon is more effectively trapped (Short and Kaldy 1995). Eelgrass collected from Great Bay for our study appears to have responded to excess nitrogen in the system with increased leaf area (Fig. 2). However, we expect that as nitrogen loading increases, plant productivity and survival will decrease along with storage (Schmidt et al. 2012; Macreadie et al. 2017; Jiang et al. 2018).

Our results also show that exposure is an important predictor of carbon storage in eelgrass meadows. The model that included both environmental and eelgrass variables to evaluate importance in meadows indicated that degree of sorting was the most important variable and was positively correlated with bulk carbon stocks. Exposure affects carbon storage through the balance of accumulation and export (Mazarrasa et al. 2018). The sites in this study have carbon sources from primarily outside the meadow, suggesting that degree of sorting in this study can be used as a measure of input of material to the site. Röhr et al. (2018) also showed positive relationships between degree of sorting and carbon stocks worldwide.

Our study identified additional environmental and plant parameters that influence C_org_ stocks. Multimodel inference showed that %silt-clay was the most important predictor of C_org_ stocks in sediment in both meadow and unvegetated sites when considering environmental variables alone. When assessing environmental and eelgrass variables in meadows, %silt-clay was still important, but was overpassed by the degree of sorting (Table 2). Results from this study support previous studies that show a positive relationship between fine-grained sediments and C_org_ stocks (e.g., Dahl et al. 2016; Röhr et al. 2016; Mazarrasa et al. 2017; Oreska et al. 2017; Röhr et al. 2018): sites with the highest C_org_ stocks (Portland, Great Bay, Boston Harbor, and Charlestown) were comprised of silt or very fine sand (Table 1; Fig. 4). The relationship between carbon storage and fine-grained sediments can be attributed to the larger surface area for adsorbing organic molecules (Bergamaschi et al. 1997) and anoxic conditions in the substrate, which allows carbon and nutrients to accumulate in soil (Koch 2001). Others have suggested that hydrodynamic regime can also explain this relationship as grain size composition of sediments is largely controlled by wave and current exposure (Mazarrasa et al. 2017; Kindeberg et al. 2018). In our study, this is supported by meadows at Charlestown where the two predictors (fine grain sediments, and low hydrodynamic energy) converge resulting in the largest eelgrass derived C_org_ stocks (Table 1).

An interesting outcome of this study is the importance of commonly measured environmental variables in predicating carbon storage in meadows, as well as unvegetated sites. In unvegetated sites, %silt-clay explained 73% of the variance in surface carbon stocks (Fig. S6b) and was not significantly different from a model including sediment trap mass, tidal range and relative wave energy. In contrast, the meadow models with environmental and eelgrass variables explained less of the variance in carbon stocks than models using environmental variables alone (Fig. 7). The difference in explained variance may be due to inclusion of variables impacting meadow health such as tidal range and insolation. It is also possible that more of the variance could have been explained if we had good measures of light attenuation (reduction in the intensity of light as it travels through water) rather than insolation (the amount of light reaching the surface of the water).

### Sources of Carbon to Seagrass Meadows

Numerous studies have reported higher C_org_ accumulation rates for seagrass sediments than predicted from plant production alone, indicating that allochthonous sources contribute to seagrass sediment C_org_ stocks (Bouillon and Boschker 2006; Kennedy et al. 2010). Kennedy et al. (2010) predicted that ~ 50% of C_org_ in seagrass sediments was of autochthonous origin. In our study, the isotope biplot suggested that eelgrass was not the dominate source of carbon in the sediment (Fig. 8). Röhr et al. (2018) found similar results in the western Atlantic where *Zostera* contributed approximately 10–40% of the carbon stock. Great Bay, Boston Harbor, and Portland have the highest C_org_ stocks, and relatively high percentage of fine-grained sediments (35%, 32%, and 28% silt-clay, respectively). However, carbon inputs to the sediments are mostly allochthonous, possibly from terrestrial processes such as sewage discharges and tributaries and/or lower water exchange with the overlying water (Koch 2001), which leads to lower oxygen concentrations that slow the decomposition rate of accumulated carbon. The other site with relatively high C_org_ stocks in the meadow, Charlestown, had a high percentage of fine-grained sediments (42%). While fine-grained sediments will most effectively bind C_org_ and reduce decomposition rates, eelgrass roots and rhizomes also have a high proportion of refractory organic compounds, and high C/N/P ratios, which makes them more resistant to degradation, relative to most marine plants and algae (Fourqurean and Schrlau 2003; Vichkovitten and Holmer 2004; Kennedy et al. 2009).

### Implications of Research

The 2017 Update of the Regional Climate Change Action Plan for the New England States and Eastern Canada Provinces (https://www.coneg.org/wp-content/uploads/transferred/Data/Sites/1/media/documents/reports/2017-rccap-final.pdf) recognizes the importance of preserving and enhancing Blue Carbon reservoirs (carbon stored in coastal mangrove, marsh and eelgrass ecosystems) to achieve CO_2_ emission reduction goals. There is little site-specific carbon sequestration data available for New England. Our data has significant value in understanding worldwide carbon budgets as well as providing input for specific region issues.

Our study also confirmed the importance of local site characteristics highlighted by other studies (Dahl et al. 2016; Jankowska et al. 2016; Macreadie et al. 2014; Mazarrasa et al. 2017, 2018; Oreska et al. 2017; Röhr et al. 2016, 2018; Samper-Villarreal et al. 2016; Serrano et al. 2016b). Most sites in this study showed the expected pattern of higher carbon stocks in meadow versus unvegetated sediments. However, at sites experiencing higher eutrophication (e.g., Portland, Great Bay), this pattern was not seen, likely because these areas are depositional, with the amount of carbon stored in the sediments driven primarily by allochthonous loading rather than by meadow characteristics. Depositional areas are known to bury carbon, although the predicted storage rate in seagrass meadows is estimated to be twice that of estuarine sediments (Duarte et al. 2005). However, the importance of blue carbon arises not only from the ability of vegetated habitats to enhance carbon storage, but also by their ability to avoid emissions from disturbed sediments (Arias-Ortiz et al. 2018b; Macreadie et al. 2018). Therefore, the importance of the high carbon storage seen in Portland and Great Bay will hinge on its stability. During the growing season, meadow vegetation alters flow and stabilizes sediments. In New England winters, eelgrass meadows senesce but because their belowground roots and rhizomes are resistant to decomposition (Mcleod et al. 2011; Dahl et al. 2016), they act to stabilize the sediments. In contrast, the unvegetated areas may be more at risk for resuspension and erosion, leading to carbon oxidation and CO_2_ release unless stabilized by macrophytes or tube-building invertebrates. Without further research to understand if this unvegetated sediment has been stabilized, it should be viewed as vulnerable to oxidation.

Loss of seagrass is predicted to result in oxidation of stored carbon, resulting in CO_2_ emissions. While previous studies have assumed 100% oxidation, more recent studies predict a 10–50% loss of carbon in the top meter of sediments (Lovelock et al. 2017; Arias-Ortiz et al. 2018b). In addition, the probability of CO_2_ release from areas with seagrass loss will be dependent on both the amount of carbon stored in the sediment and the probability of carbon mineralization due to oxidation (Lovelock et al. 2017) so preservation of existing meadows should be prioritized. Once eelgrass meadows are lost, decreased sediment stability, increased turbidity, decreased water clarity, and increased phytoplankton blooms can decrease the probability of natural eelgrass recovery (Unsworth et al. 2015). Disturbances should be minimized as they can adversely impact seagrass and decrease carbon storage (Baraňano et al. 2018; Macreadie et al. 2015; Serrano et al. 2016a, b; Trevathan-Tackett et al. 2017b). However, restoration or regrowth of seagrass has been shown to restore the meadow’s capacity for carbon storage (Macreadie et al. 2015; Marba et al. 2015). Given that eelgrass seeds are only viable for 1 year (Jarvis and Moore 2010; Orth et al. 2000), and the plants have specific water quality requirements (Dennison et al. 1993), additional management actions to stabilize the sediment and reintroduce seagrass may be needed to reestablish existing beds. This study also suggests that measurement of environmental variables that impact both seagrass meadow persistence and carbon storage may be a useful proxy to predict carbon stores.

## Supplementary Material

Supplement1

Supplement2

## Figures and Tables

**Table 1:** Site characteristics. Sites are arranged from north to south. Relative exposure is based on the amount of enclosure at the site and fetch at eelgrass sites. Tidal ranges were obtained from NOAA datums (https://tidesandcurrents.noaa.gov/stations.html?type=Datums). The average salinity and range (in brackets) were obtained from the US EPA’s National Coastal Assessment Program (http://www.epa.gov/emap). Substrate is the median grain size from the top 5 cm of the meadow sediment cores from this study, summarized as the appropriate Wentworth class

Site	Latitude (dd)	Longitude (dd)	Relative exposure	Tidal range (m)	%Nitrogen in eelgrass	Salinity (psu)	Substrate
Portland, ME	43.67	− 70.24	Moderate	2.78–3.02	2.44	30.8(29.0–31.7)	Very fine sand
Great Bay, NH	43.07	− 70.88	Moderate	2.05–2.25	1.74	25.9(16.5–30.9)	Silt
Gloucester, MA	42.60	− 70.66	Low	2.68–2.92	1.13	31.6(30.1–33.6)	Medium sand
Nahant, MA	42.42	− 70.92	Moderate	2.89–3.13	1.08	31 (30.2–31.4)	Fine sand
Boston Harbor, MA	42.33	− 70.96	Moderate	2.89–3.13	1.56	30.3 (24.6–32.9)	Very fine sand
Cohasset, MA	42.25	− 70.78	High	2.89–3.13	1.18	30.3 (29.8–30.8)	Fine sand
Orleans, MA	41.75	− 69.95	Low	1.20–1.40	1.32	30.7(30.2–31.5)	Very fine sand
W. Falmouth, MA	41.61	− 70.65	Low	0.55–0.67	0.93	30.1 (29.5–31.0	Medium sand
Prudence Island, RI	41.58	− 71.32	High	1.14–1.26	1.71	30.4 (19.5–32.9)	Medium sand
Martha’s Vineyard, MA	41.46	− 70.60	Moderate	0.85–0.95	0.97	28.7(18.1–31.8)	Fine sand
Charlestown, RI	41.37	− 71.64	Low	0.77–0.88	1.16	29.6 (26.0–32.3)	Silt

**Table 2:** Importance of individual variables to predicting the carbon stock in the top 5 cm of the core. ∑AICω near 1 indicate high importance while those near zero have low importance

Model	∑AICω	Environmental variables only	∑AICω	Environmental and eelgrass variables
Meadow	0.77	%Silt-clay	0.82	Degree of sorting
	0.39	%N in sediment trap	0.74	Shoot density (/m^2^)
	0.28	Degree of sorting	0.44	%Silt-clay
	0.27	Tidal range (m)	0.16	Tidal range (m)
	0.25	Insolation (kW h/m^2^/day)	0.12	Growth (mg/shoot/day)
	0.22	Sediment trap mass (g)		
Unvegetated	1.00	%Silt-clay		N/A
	0.22	Sediment trap mass (g)		
	0.22	Tidal range (m)		
	0.16	Relative wave energy		

**Table 3:** Sediment accumulation and carbon accumulation based on 210Pb modeling from sediment cores. In some cases (Portland, Nahant), sedimentation rates could be determined, but uncertainties in the model did not allow a definitive core date to be defined

Site	Site code	Sediment accumulation rate (g/cm^2^/year)	Carbon accumulation rate (g C/m^2^/year)	Core age (year)
Portland, ME	PO	0.16 ± 0.09	48 ± 25	No data^c^
Great Bay, NH	GB	1.19 ± 0.15	230 ± 30	18
Gloucester, MA	NB	0.121 ± 0.008	16.7 ± 1.1	113
Nahant, MA	PC	0.29 ± 0.08	20 ± 5	No data^c^
Boston Harbor, MA	BH	No data^a^	No data^a^	No data^a^
Cohasset, MA	CT	No data^a^	No data^a^	No data^a^
Orleans, MA	PB	0.27 ± 0.08	8 ± 2	95
W. Falmouth, MA	WF	No data^b^	No data^b^	No data^b^
Prudence Island, RI	PI	0.14 ± 0.02	8.9 ± 1.2	113
Martha’s Vineyard, MA	MV	No data^a^	No data^a^	No data^a^
Charlestown, RI	NP	0.26 ± 0.03	89 ± 10	54

a Signs of disturbance/mixing in profile

b Sediment accumulation low or negligable

c Age could not be calculated

**Table 4:** Comparison of carbon inventory from this study to other areas

Study	Geographic area	Seagrass type	Seagrass aboveground C (g C/m^2^)	Sediment C (g C/m^2^)
Fourqurean et al. (2012) ^a^	Worldwide	All species	251 ± 49	19,420 ± 202
	North Athlantic	All species	85 ± 19	4870 ± 1450
Röhr et al. (2018)^b^	Worldwide Western Atlantic	*Zostera marina* *Zostera marina*		2721 ± 989 1349 ± 2
This study^b^	Northwestern Atlantic	*Zostera marina*	86 ± 19	2832 ± 416

a Reported as mean ± 95% confidence interval

b Reported as mean ± standard error
