# Supplementary material for: Factors Influencing Carbon Stocks and Accumulation Rates in Eelgrass Meadows Across New England, USA
Source: Estuaries Coast. Author manuscript; Available in PMC 2021 Dec 1. (PMC7751660)
Supplement: Supplement1 [file NIHMS1642087-supplement-Supplement1.pptx]

## Slide 1
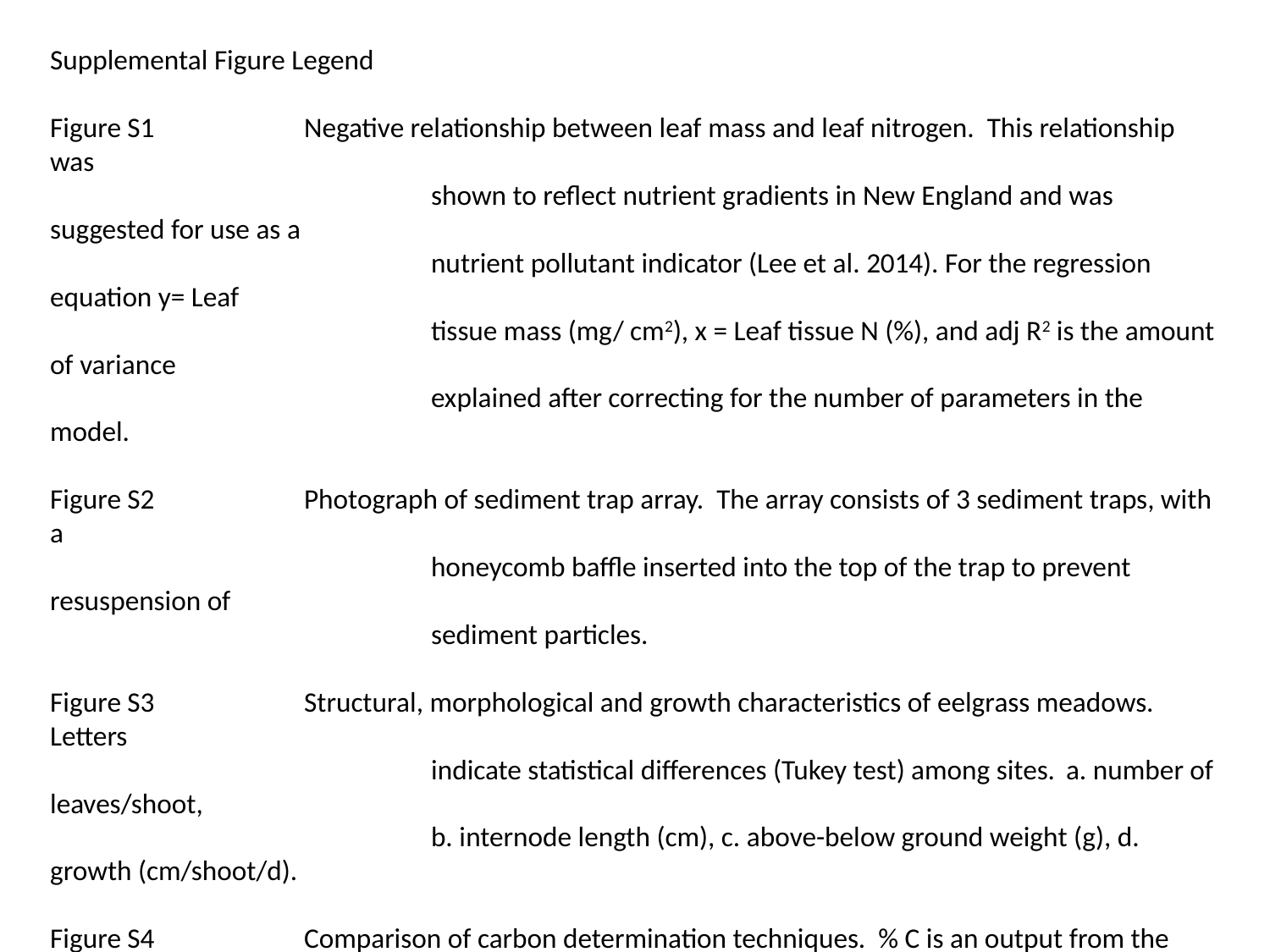

Supplemental Figure Legend
Figure S1		Negative relationship between leaf mass and leaf nitrogen. This relationship was
			shown to reflect nutrient gradients in New England and was suggested for use as a
			nutrient pollutant indicator (Lee et al. 2014). For the regression equation y= Leaf
			tissue mass (mg/ cm2), x = Leaf tissue N (%), and adj R2 is the amount of variance
			explained after correcting for the number of parameters in the model.
Figure S2		Photograph of sediment trap array. The array consists of 3 sediment traps, with a
			honeycomb baffle inserted into the top of the trap to prevent resuspension of
			sediment particles.
Figure S3		Structural, morphological and growth characteristics of eelgrass meadows. Letters
			indicate statistical differences (Tukey test) among sites. 	a. number of leaves/shoot,
			b. internode length (cm), c. above-below ground weight (g), d. growth (cm/shoot/d).
Figure S4		Comparison of carbon determination techniques. % C is an output from the
			isotope-ratio mass spectrometer. Organic matter is quantified using the loss on
			ignition technique.
Figure S5		Carbon stock (g C / m2) of material collected in sediment traps in meadow and
			unvegetated areas. Capital letters indicate statistical differences (Tukey test) among
			sites in meadows, while lower case letters indicate statistical differences (Tukey test)
			among sites in unvegetated areas.

## Slide 2
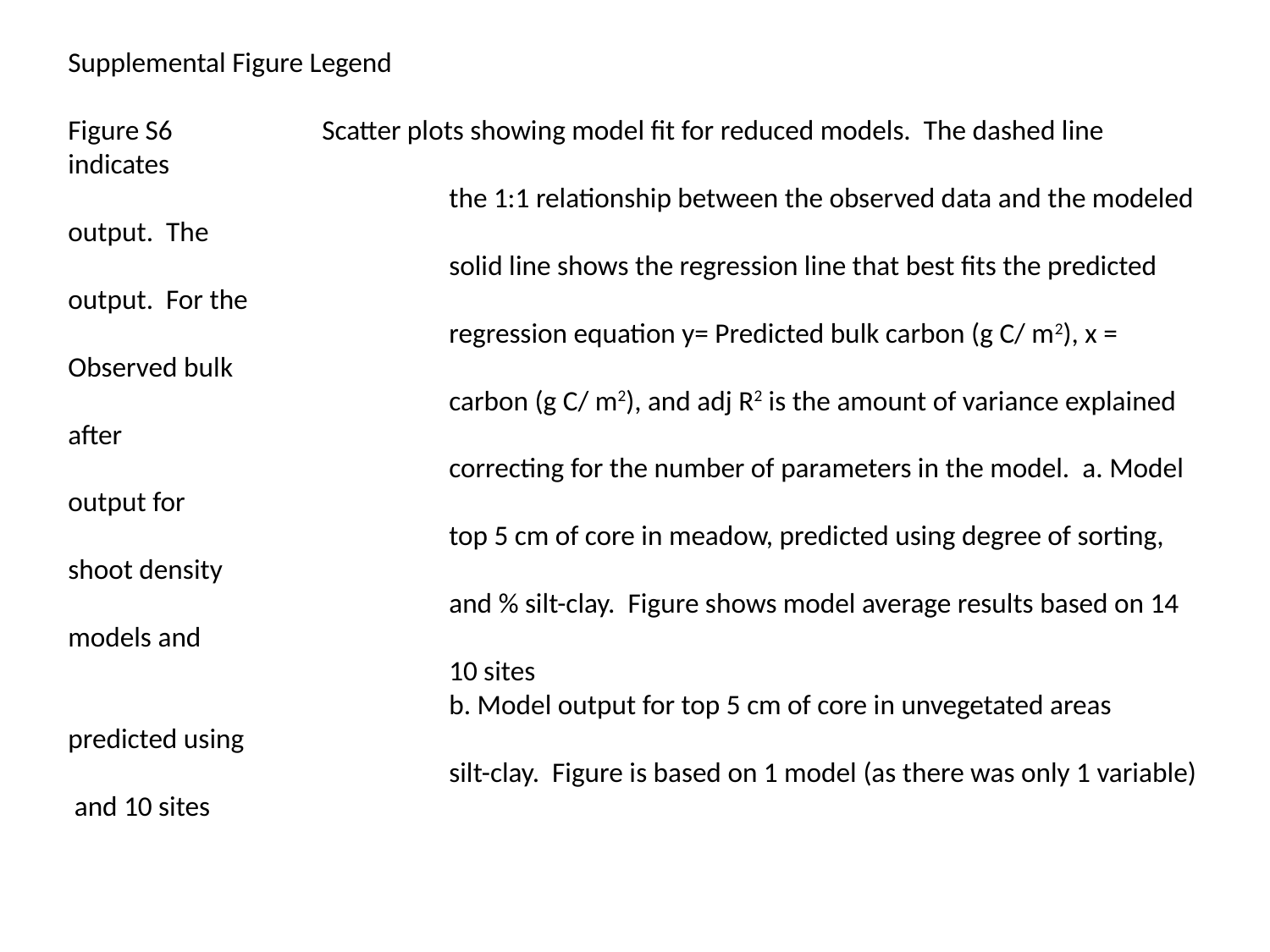

Supplemental Figure Legend
Figure S6		Scatter plots showing model fit for reduced models. The dashed line indicates
			the 1:1 relationship between the observed data and the modeled output. The
			solid line shows the regression line that best fits the predicted output. For the
			regression equation y= Predicted bulk carbon (g C/ m2), x = Observed bulk
			carbon (g C/ m2), and adj R2 is the amount of variance explained after
			correcting for the number of parameters in the model. a. Model output for
			top 5 cm of core in meadow, predicted using degree of sorting, shoot density
			and % silt-clay. Figure shows model average results based on 14 models and
			10 sites
			b. Model output for top 5 cm of core in unvegetated areas predicted using
			silt-clay. Figure is based on 1 model (as there was only 1 variable) and 10 sites

## Slide 3
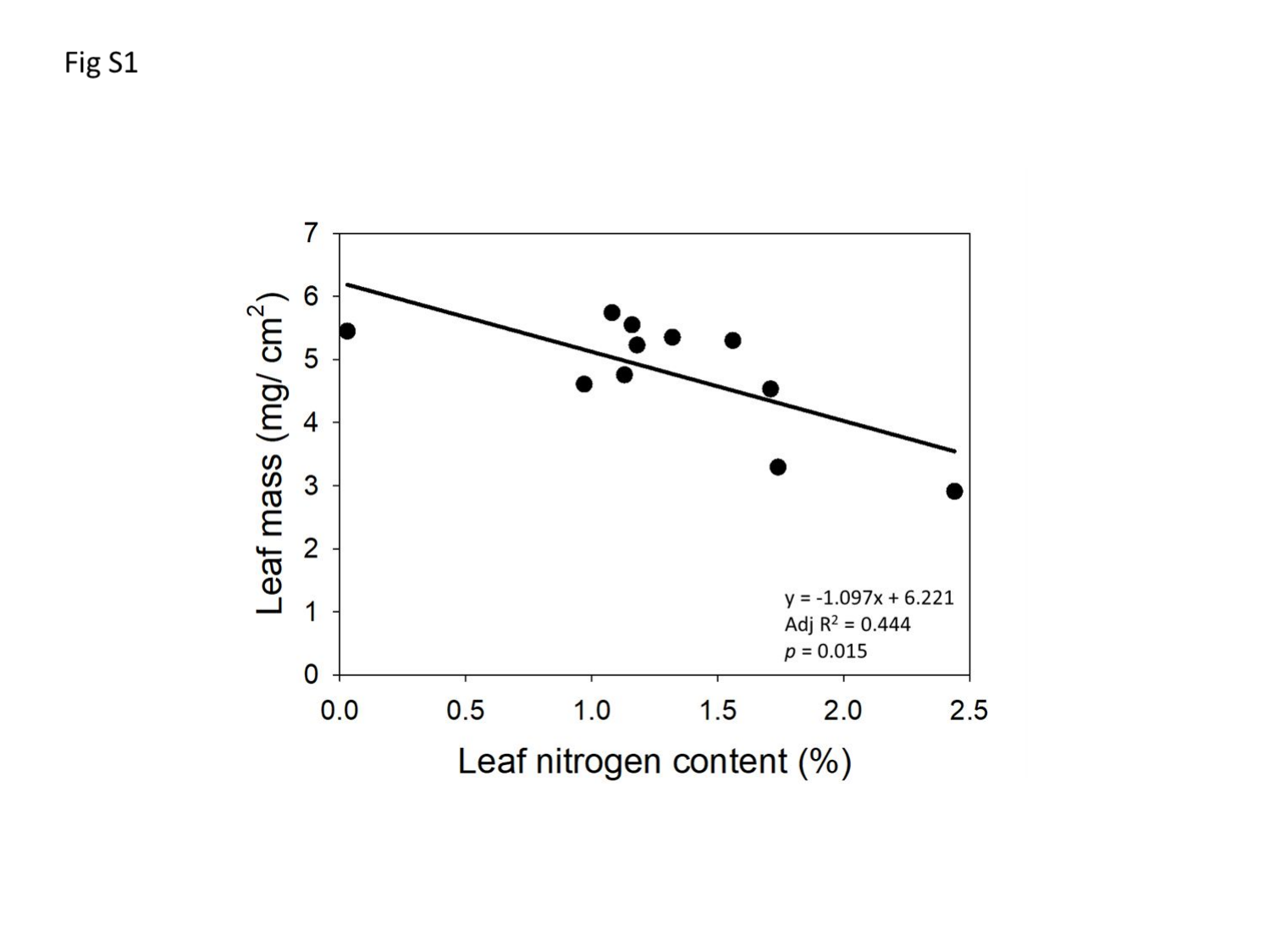

## Slide 4
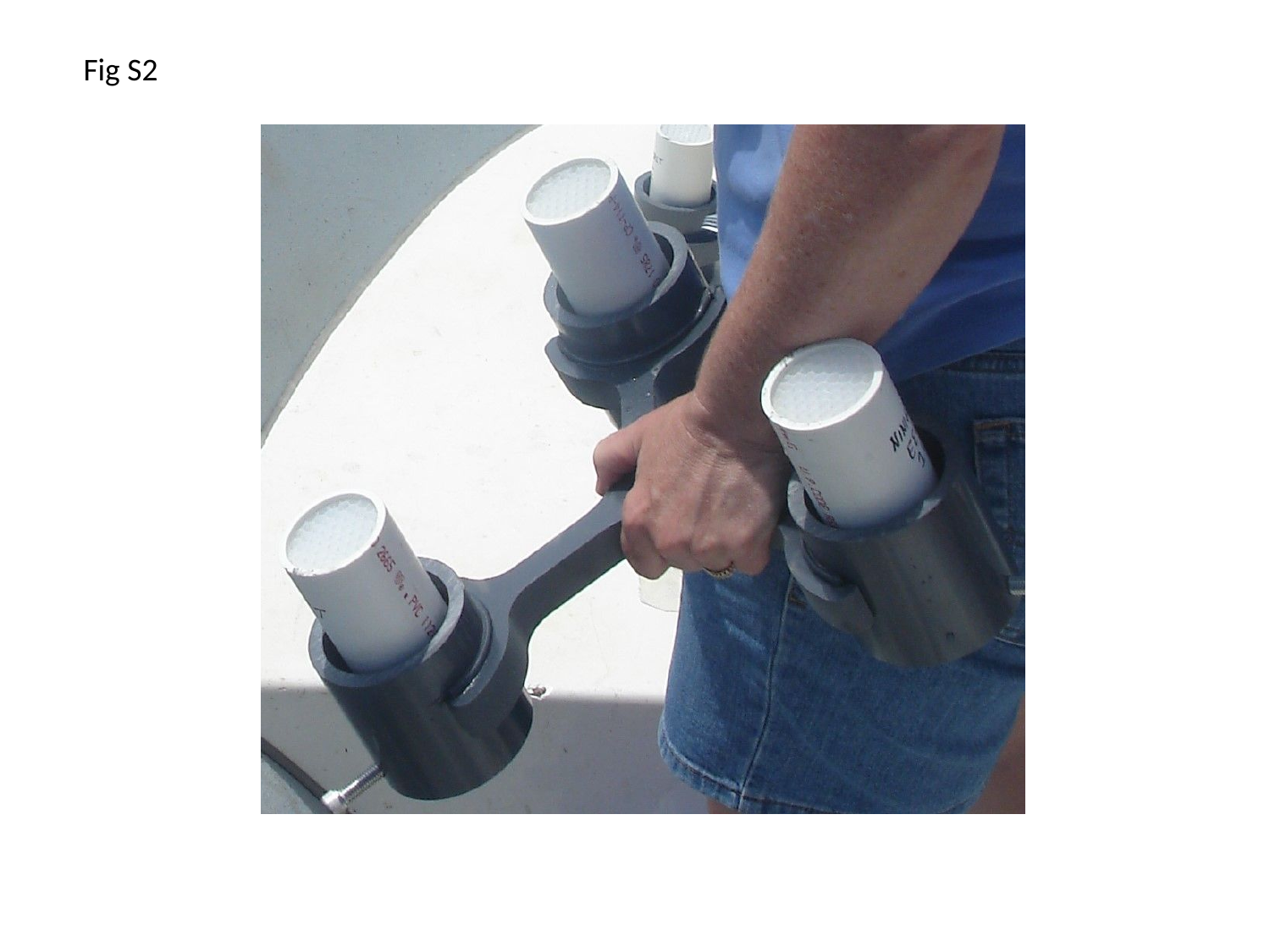

Fig S2

## Slide 5
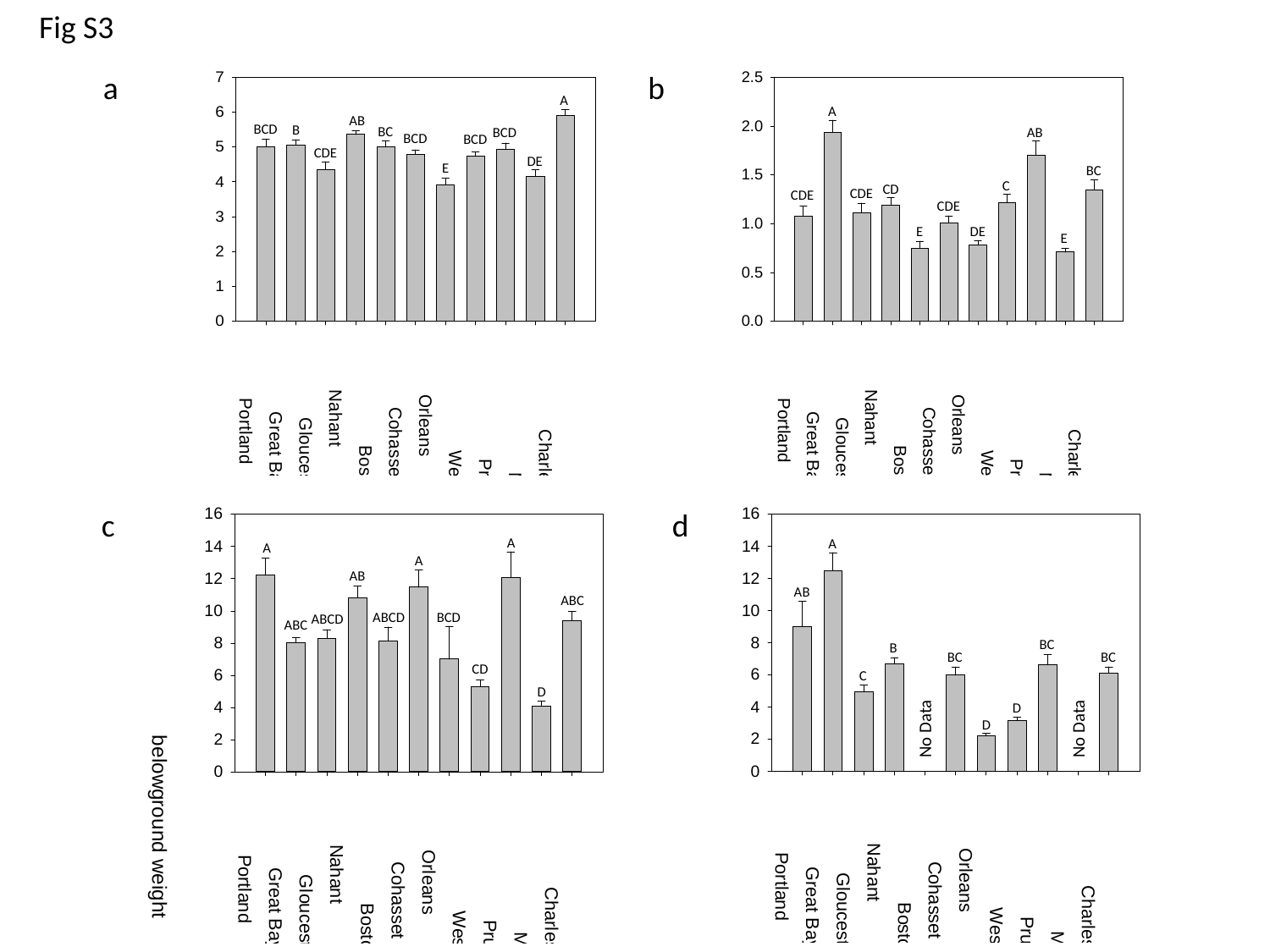

Fig S3
A
AB
BCD
B
BC
BCD
BCD
BCD
CDE
DE
E
A
AB
BC
C
CD
CDE
CDE
CDE
E
DE
E
a
b
A
A
A
AB
ABC
BCD
ABCD
ABCD
ABC
CD
D
A
AB
BC
B
BC
BC
C
D
D
No Data
No Data
c
d

## Slide 6
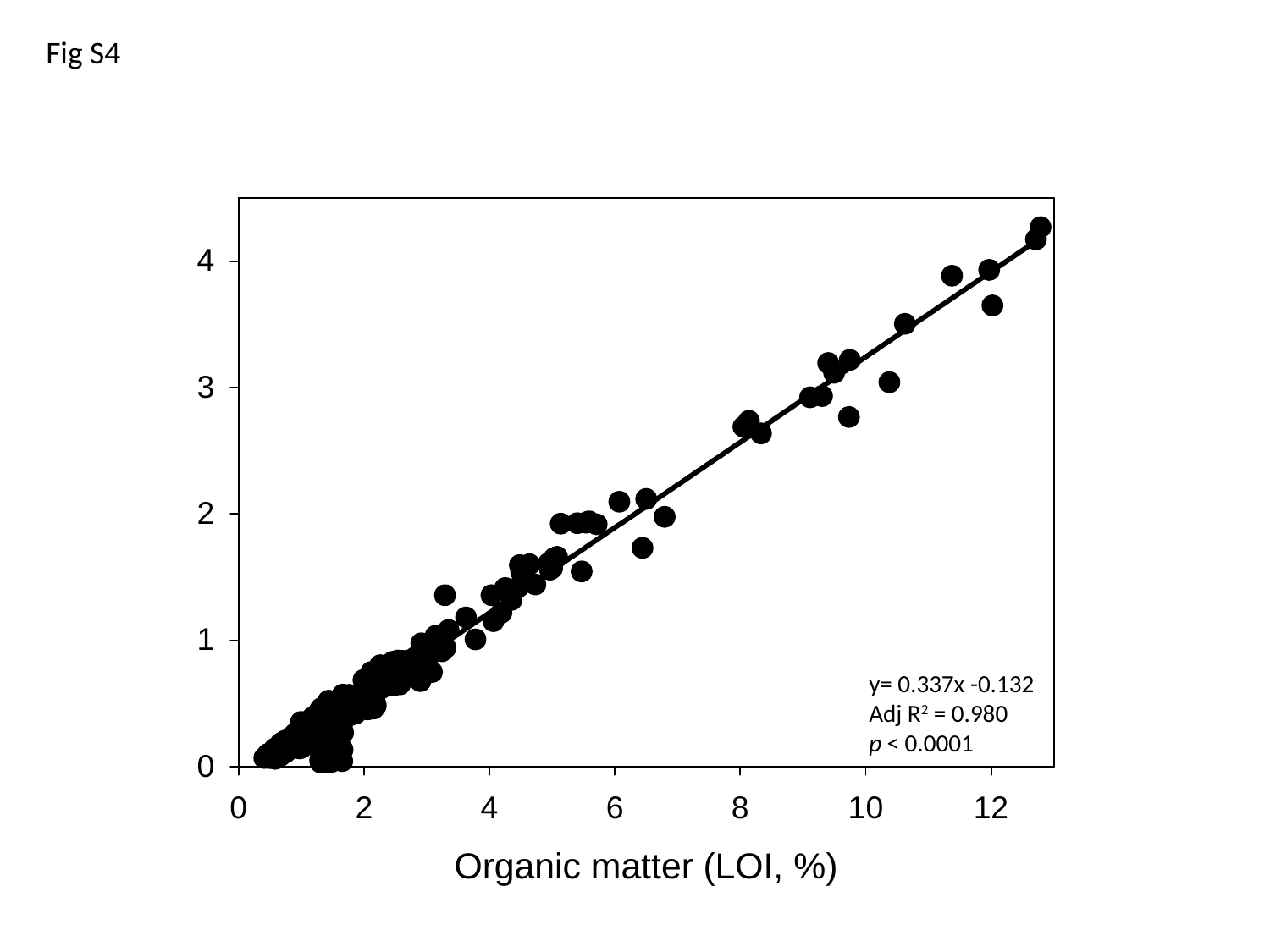

Fig S4
y= 0.337x -0.132
Adj R2 = 0.980
p < 0.0001

## Slide 7
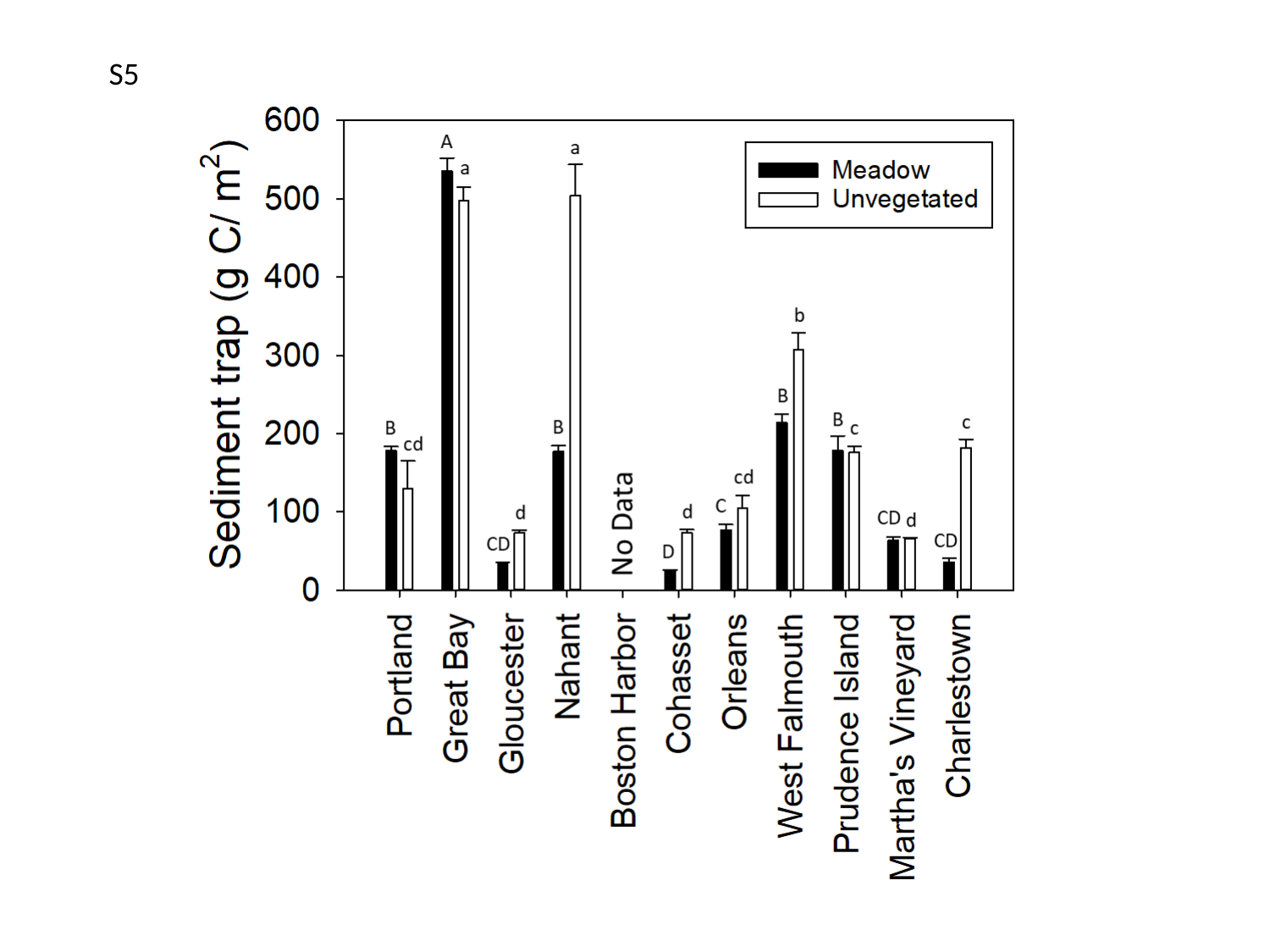

S5

## Slide 8
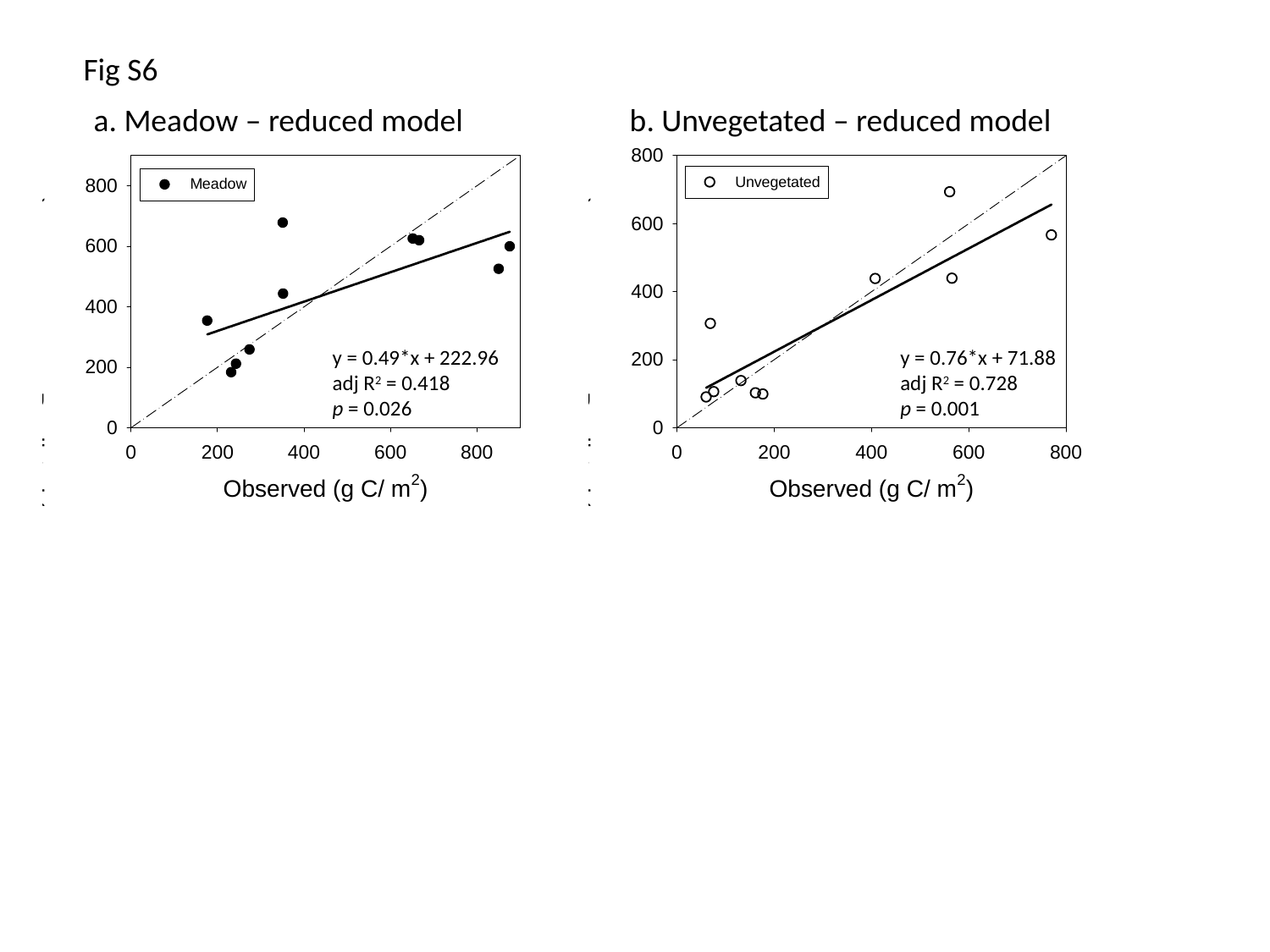

Fig S6
a. Meadow – reduced model
b. Unvegetated – reduced model
y = 0.49*x + 222.96
adj R2 = 0.418
p = 0.026
y = 0.76*x + 71.88
adj R2 = 0.728
p = 0.001
